# Systematic pan-cancer analysis reveals context-dependent prognostic and immunological roles of WDR1 with divergent effects in renal and gastric cancers

**DOI:** 10.3389/fimmu.2026.1864405

**Published:** 2026-06-30

**Authors:** Qiu-shuang Li, Song-xin Tang, Xin-yi Li, Ying-chao Shi, Feng-yun Ru, Jin-hui Li, Yong-zhu Wang, Chen-xi Zhang, Bai-yin Yuan

**Affiliations:** 1 College of Life and Health Sciences, Wuhan University of Science and Technology, Wuhan, China; 2 Central China Subcenter of National Center for Cardiovascular Diseases, Henan Cardiovascular Disease Center, Fuwai Central-China Cardiovascular Hospital, Central China Fuwai Hospital of Zhengzhou University, Zhengzhou, China; 3 Central Laboratory, Nanjing Chest Hospital, Affiliated Nanjing Brain Hospital of Nanjing Medical University, Nanjing, China

**Keywords:** biological function, immune infiltration, pan-cancer, prognosis, WDR1

## Abstract

**Background:**

WD-repeat domain 1 (WDR1), a key regulator of actin cytoskeleton dynamics, has been implicated in tumor progression. However, its pan-cancer characteristics and immunological relevance remain unclear. In this study, we performed an integrative pan-cancer analysis to evaluate the expression patterns, prognostic value, molecular features, and immune associations of WDR1.

**Methods:**

We systematically analyzed WDR1 across cancers using integrated datasets from The Cancer Genome Atlas (TCGA), Genotype-Tissue Expression (GTEx), and Gene Expression Omnibus (GEO). By integrating R software and online bioinformatics platforms (e.g., UALCAN, HPA, GEPIA2, cBioPortal, and TIMER2.0), we characterized WDR1 in terms of expression, prognostic and diagnostic value, epigenetic regulation, genetic variation, and immune cell infiltration. Functional enrichment and drug sensitivity analyses were further conducted, and *in vitro* experiments were performed for validation.

**Results:**

WDR1 exhibited heterogeneous expression across cancers and showed tumor-type–dependent prognostic significance. In addition, WDR1 displayed differential DNA methylation patterns and was positively correlated with multiple RNA methylation–related regulators. Notably, WDR1 expression was closely associated with tumor immune microenvironment features, including increased infiltration of myeloid and stromal cells and broad correlations with immune checkpoint molecules and immune-related genes. Functional analyses indicated that WDR1-related genes were primarily involved in actin cytoskeleton organization. Drug sensitivity analysis revealed that WDR1 expression was associated with differential responses to multiple therapeutic agents. Importantly, experimental validation demonstrated that WDR1 exerted context-dependent biological effects, suppressing proliferation, migration, and invasion in renal cancer cells while promoting these phenotypes in gastric cancer cells.

**Conclusion:**

Our findings demonstrated that WDR1 was associated with tumor progression and immune microenvironment remodeling in a context-dependent manner and may represent a potential biomarker for further investigation in cancer.

## Introduction

Cancer remains one of the leading causes of mortality worldwide and continues to pose a formidable public health challenge (1–4). While immunotherapy and targeted therapy have revolutionized the oncological landscape as novel therapeutic strategies (5), the five-year survival rate for many patients remains suboptimal, and the clinical efficacy of immunotherapy requires further optimization (3, 6). A defining characteristic of cancer is its substantial heterogeneity across different malignancies in terms of incidence, mortality, and underlying molecular drivers; yet, many cancers share conserved signaling pathways and potential therapeutic targets (7, 8). Therefore, identifying effective and broadly applicable biomarkers, particularly those involved in fundamental cellular processes, is crucial for improving cancer diagnosis and treatment outcomes.

Actin dynamics—the simultaneous and coordinated polymerization and depolymerization of actin filaments—is fundamental to a range of biological processes, including cell proliferation, migration, morphogenesis, and cell polarity establishment (9–11). Disruptions in actin dynamics are closely associated with various pathological conditions, particularly cancer, as well as neurological and immunological disorders (12–15). Actin dynamics is tightly orchestrated by a variety of actin-binding proteins (13, 16), among which the Actin Depolymerizing Factor (ADF)/cofilin family plays a central role by inducing filament severing and monomer dissociation (17). WDR1 (WD-repeat domain 1), the mammalian homolog of AIP1 (actin-interacting protein 1) in Drosophila, acts as a critical cofactor for ADF/cofilin, significantly accelerating actin depolymerization by capping the barbed ends of ADF/cofilin-bound filaments (18, 19). As a highly conserved protein, WDR1 participates in multiple actin-dependent processes, including cell division, migration, and epithelial polarity establishment and maintenance (20–25).

The proliferative, migratory, and invasive properties of tumor cells rely heavily on dynamic cytoskeletal remodeling. Increasing evidence indicates that actin dynamics contributes not only to tumor initiation and progression but also to the modulation of immune responses within the immunological synapse (26–28). As a key regulator of actin dynamics, WDR1 has been increasingly recognized for its involvement in tumorigenesis. Aberrant expression of WDR1 has been reported in several malignancies. For instance, elevated WDR1 expression has been observed in thyroid tumors and glioblastoma and is associated with invasive phenotypes (29, 30). Proteomic analyses have identified higher WDR1 levels in ovarian carcinoma compared with normal tissue, suggesting its potential as a therapeutic target (31). Similarly, WDR1 overexpression has been linked to enhanced migration and poor prognosis in breast cancer (32).

Our previous studies further demonstrated that WDR1 was significantly upregulated in non–small cell lung cancer (NSCLC) tissues and was associated with unfavorable clinical outcomes. Mechanistically, WDR1 promoted tumor cell proliferation, migration, and invasion through ADF/cofilin-mediated actin depolymerization and regulated YAP signaling via cytoskeleton-dependent cortical tension (33, 34). In addition, WDR1 has been reported to facilitate tumor progression in hepatocellular carcinoma, pancreatic ductal adenocarcinoma, and prostate cancer, partly through pathways such as Wnt/β-catenin signaling (35–37). However, the biological role of WDR1 appears to be context-dependent and, in some instances, controversial. Notably, in osteosarcoma, conflicting studies have identified WDR1 as either a protective factor that was significantly downregulated (38) or a promoter of osteosarcoma progression (39). However, current studies on WDR1 have primarily focused on individual cancer types, and a comprehensive understanding of its expression heterogeneity across cancers, as well as its potential associations with clinical features and regulatory mechanisms, remains limited.

In this study, we performed a systematic pan-cancer analysis centered on The Cancer Genome Atlas (TCGA), Genotype-Tissue Expression (GTEx), and Gene Expression Omnibus (GEO) databases, complemented by online bioinformatics platforms, to characterize the expression patterns, prognostic value, genomic alterations, and single-cell distribution of WDR1 across diverse cancer types. Furthermore, we explored its associations with immune cell infiltration, immune-related genes, immune checkpoints, methylation status, tumor stemness, and drug sensitivity. Functional enrichment analyses were conducted to investigate potential molecular mechanisms. In addition, we performed experimental validation in kidney renal clear cell carcinoma (KIRC) and stomach adenocarcinoma (STAD) to further elucidate the biological role of WDR1. In summary, these findings provide a comprehensive overview of WDR1 and support its potential as a prognostic biomarker and therapeutic target in cancer.

## Materials and methods

### Gene expression analysis of WDR1 in pan-cancer

The Human Protein Atlas (HPA) database (https://www.proteinatlas.org/) was used to assess the baseline expression of WDR1 in normal tissues. Immunohistochemical images of WDR1 protein expression in normal and tumor tissues were obtained from the Tissue and Pathology modules, and immunofluorescence images for the subcellular localization of WDR1 were retrieved from the Subcell module. TIMER2.0 (http://timer.cistrome.org/) was used to compare WDR1 expression between tumor and adjacent normal tissues across TCGA cancers using the Gene_DE module (40).

To validate WDR1 expression at the protein level, quantitative proteomic data and corresponding clinical annotations for the CPTAC KIRC cohort were downloaded from the CPTAC database. Protein abundance values were represented as log2 ratios relative to a pooled reference sample (POOL). WDR1 protein expression levels were extracted and matched with sample annotation information. Samples without paired tumor–normal annotations were excluded. Ultimately, 81 matched tumor–normal pairs were included for analysis. Differences in WDR1 protein abundance between paired tissues were evaluated using the paired Wilcoxon signed-rank test. Data processing and visualization were performed in R software, with a two-sided P < 0.05 considered statistically significant.

A uniformly processed pan-cancer dataset was downloaded from the UCSC Xena platform (https://xenabrowser.net/datapages/). WDR1 expression across 33 cancer types was extracted and transformed as log2 (TPM + 1). Differences between tumor and normal tissues were evaluated using the Wilcoxon rank-sum test, with P < 0.05 considered statistically significant. All data analyses were performed using R software (version 4.0.2), and visualization was conducted using the ggplot2 R package.

### Prognostic and diagnostic value of WDR1

Clinical and gene expression data for pan-cancer samples were obtained from the UCSC Xena platform. Samples with available survival information were included in the prognostic analysis. Overall survival (OS), disease-specific survival (DSS), and progression-free interval (PFI) were used as clinical endpoints to assess the prognostic value of WDR1 across cancers. Univariate Cox regression analysis was performed to calculate hazard ratios (HRs) and 95% confidence intervals (CIs) for each cancer type. Patients were divided into high- and low-expression groups according to the median WDR1 expression, and Kaplan–Meier survival analysis was conducted to compare survival outcomes between groups.

For diagnostic analysis, WDR1 expression data in tumor and normal tissues were extracted from the integrated TCGA and GTEx datasets. Receiver operating characteristic (ROC) curve analysis was performed to evaluate the diagnostic value of WDR1, and the area under the curve (AUC) was calculated. Statistical analyses were conducted using R software (version 4.0.2) with P < 0.05 considered statistically significant.

### DNA and RNA methylation-related analysis of WDR1

The UALCAN database (https://ualcan.path.uab.edu/index.html) was used to compare the promoter methylation levels of WDR1 between tumor and normal tissues across cancer types. The prognostic value of WDR1 methylation was further evaluated using the Gene Set Cancer Analysis (GSCALite) platform (https://guolab.wchscu.cn/GSCA/), including its associations with disease-free interval (DFI), disease-specific survival (DSS), overall survival (OS), and progression-free survival (PFS).

For RNA methylation-related analysis, pan-cancer expression data were obtained from UCSC Xena platform and only tumor samples were included. Expression data of WDR1 and 44 RNA methylation regulators (m1A, m5C, and m6A), were extracted for each cancer type. Pearson correlation analysis was performed to assess the associations between WDR1 expression and these regulators across cancers. Data processing and visualization were performed using R software.

### Genetic alteration and copy number variation analysis of WDR1

The cBioPortal database (https://www.cbioportal.org/) was used to analyze the genetic alteration profile of WDR1 across the TCGA Pan-Cancer Atlas Studies, including alteration frequency and amplification events. For copy number variation (CNV) analysis, gene expression data and GISTIC-processed gene-level CNV data were obtained from the UCSC Xena platform and matched by sample ID. Within each cancer type, samples were categorized into gain, neutral, and loss groups based on GISTIC CNV status, and differences in WDR1 expression among groups were compared using the Kruskal–Wallis test.

### Immune-related characteristics of WDR1 in pan-cancer

TIMER2.0 was utilized to evaluate the association between WDR1 expression and immune cell infiltration across TCGA cancers using multiple algorithms (TIMER, CIBERSORT, quanTIseq, xCell, MCP-counter, and EPIC). Additionally, TIDE analysis was performed. This analysis covered a broad range of immune cell subsets, including T cells, B cells, macrophages, natural killer (NK) cells, dendritic cells, neutrophils, regulatory T cells (Tregs), and cancer-associated fibroblasts.

In addition, pan-cancer transcriptomic data from the UCSC Xena platform were used to analyze the associations between WDR1 expression and immune-related genes. A total of 150 immune-related genes, including chemokines, receptors, major histocompatibility complex (MHC) molecules, immunoinhibitors, and immunostimulators, were included. Spearman correlation analysis was performed to assess these associations across cancer types, and the results were visualized as pan-cancer heatmaps using R software.

### Single-cell functional analysis and tumor biological features of WDR1

Single-cell expression patterns of WDR1 across tumor microenvironment cell populations in pan-cancer datasets were systematically analyzed using the Tumor Immune Single-cell Hub (TISCH) database (https://tisch.compbio.cn/home/) (41). The CancerSEA database was further used to evaluate the associations between WDR1 expression and multiple functional states of tumor cells at the single-cell level, including proliferation, apoptosis, cell cycle, epithelial–mesenchymal transition (EMT), invasion, metastasis, and stemness (42).

TCGA pan-cancer gene expression data were used to assess the associations between WDR1 expression and tumor biological features, including tumor mutation burden (TMB), microsatellite instability (MSI), DNA stemness score (DNAss), and RNA stemness score (RNAss), using correlation analysis.

### Co-expression and enrichment analysis of WDR1

A protein–protein interaction (PPI) network centered on WDR1 was constructed using the Search Tool for the Retrieval of Interacting Genes/Proteins (STRING) database (https://cn.string-db.org/), and 50 WDR1-interacting proteins were identified. The Similar Genes Detection module of Gene Expression Profiling Interactive Analysis 2 (GEPIA2) (http://gepia2.cancer-pku.cn/) was used to identify genes co-expressed with WDR1 based on TCGA tumor and normal tissue datasets, and the top 100 WDR1-related genes were retrieved (43). The integrated gene set from STRING and GEPIA2 was used for subsequent analysis. Pearson correlation analysis was then performed to evaluate the associations between WDR1 and these genes, and the top 10 positively correlated genes were selected. Their correlations with WDR1 were visualized using GEPIA2 scatter plots.

Gene Ontology (GO) and Kyoto Encyclopedia of Genes and Genomes (KEGG) pathway enrichment analyses were performed for the integrated gene set. Transcription factor enrichment analysis was further conducted using the Metascape database based on the same gene set (44). In addition, the promoter sequence of WDR1 was obtained from NCBI database, and potential transcription factor binding sites were predicted using the Joint Project on the Collection and Analysis of Promoters of Eukaryotic Genes (JASPAR) database (https://jaspar.elixir.no/).

### Gene set enrichment analysis and drug sensitivity analysis of WDR1

GSEA was performed to identify pathways associated with WDR1 expression in STAD and KIRC (45). Tumor samples were divided into high- and low-expression groups based on the median WDR1 expression. Genes were ranked based on log2 fold change (log2FC) between the two groups, and preranked GSEA was performed using KEGG and Hallmark gene sets from the Molecular Signatures Database (MSigDB). Enrichment results were evaluated using the normalized enrichment score (NES), and the top 5 enriched pathways ranked by NES were selected for visualization. A false discovery rate (FDR) q-value < 0.25 was considered statistically significant.

Drug sensitivity analysis was performed using the GSCALite platform, which integrated drug response data from the Genomics of Drug Sensitivity in Cancer (GDSC) database. The association between WDR1 mRNA expression and drug sensitivity was evaluated using Spearman correlation analysis, with drug response quantified by the half-maximal inhibitory concentration (IC50). A positive correlation indicates that higher WDR1 expression is associated with drug resistance (higher IC50), whereas a negative correlation indicates increased sensitivity (lower IC50). Multiple testing correction was applied, and FDR < 0.05 was considered statistically significant.

### Cell lines, cell culture, protein overexpression, and lentiviral shRNA-mediated knockdown

The human normal renal epithelial cell line HK-2, renal cancer cell lines 786-O, 769-P and ACHN, the human normal gastric epithelial cell line GES-1, and gastric cancer cell lines AGS, SGC-7901, HGC-27 and MGC-803 were obtained from the Cell Bank of the Chinese Academy of Sciences (Shanghai, China). All cells were cultured in their respective recommended media supplemented with 10% fetal bovine serum (FBS; Gibco, USA) and 1% penicillin-streptomycin and maintained at 37 °C in a humidified atmosphere containing 5% CO_2_.

To establish stable WDR1 knockdown, lentiviral vectors expressing shRNAs targeting WDR1 were constructed using the pLKO.1 vector (46). The target sequences were as follows: shWDR1-1, 5′-GCTGGGAAGATCAAAGACATT-3′; shWDR1-2, 5′-GCAGTTTAAGAAGTGGCAGAA-3′. A non-targeting shRNA targeting GFP (5′-GCAAGCTGACCCTGAAGTTCAT-3′) was used as the control (shCTL). For overexpressing of WDR1, WDR1 cDNA was cloned into pcDNA3.1-GFP to construct overexpressed plasmid, and the empty vector was used as a negative control.

### Western blot

Total protein was extracted using cell lysis buffer (Beyotime, China) supplemented with protease inhibitors. Equal amounts of protein were separated by 10% SDS-PAGE and transferred onto PVDF membranes. After blocking, membranes were incubated with primary antibodies against WDR1 (1:1000, 13676-1-AP, Proteintech, China) and GAPDH (1:2000, AP0063, Bioworld Technology, China), followed by the corresponding secondary antibodies. Protein bands were visualized using an enhanced chemiluminescence kit (Beyotime, China). Protein levels normalized to GAPDH were quantified using ImageJ software.

### Cell proliferation assay

Cell proliferation was assessed using the Cell Counting Kit-8 (CCK-8) assay. Cells were seeded into 96-well plates at an appropriate density and cultured for the indicated time points. At each time point, 10 μL of CCK-8 reagent was added to each well and incubated according to the manufacturer’s instructions. Each experiment was repeated independently in quadruplicate.

### Wound healing assay

Cells were seeded into 6-well plates and cultured to approximately 90% confluence, followed by 24 hours serum starvation. A linear wound was generated using a 200-μL pipette tip, and detached cells were removed with PBS. Cells were then maintained in serum-free medium, and images were captured at indicated time points using an inverted microscope (IX81, Olympus, Japan). Wound closure was quantified using ImageJ.

### Transwell invasion assay

Transwell invasion assay was assessed using a 24-well Transwell chamber (8-μm pore size; Corning, USA) pre-coated with diluted Matrigel (1:2, BD Biosciences). Cells suspended in 100 μL serum-free medium were seeded into the upper chamber, while the lower chamber was filled with medium containing 10% FBS. After 48 hours of incubation, cells remaining in the upper chamber were removed with cotton swabs. Invaded cells on the lower surface of the membrane were fixed with 4% formaldehyde and stained with crystal violet. Images were captured, and invading cells were quantified.

### Statistical analysis

Data were presented as mean ± SEM (standard error of the mean). Statistical analyses were performed using GraphPad Prism or equivalent statistical software. Comparisons between two groups were conducted using an unpaired Student’s t-test, while comparisons among multiple groups were performed using one-way ANOVA followed by Tukey’s *post hoc* test. For data that did not meet normality assumptions, non-parametric tests were applied. Cell viability curves were analyzed using two-way ANOVA with time and treatment as variables. Statistical significance was defined as *P < 0.05, **P < 0.01, ***P < 0.001, and ****P < 0.0001.

## Results

### Expression patterns of WDR1 across cancers

**Figure 1:**
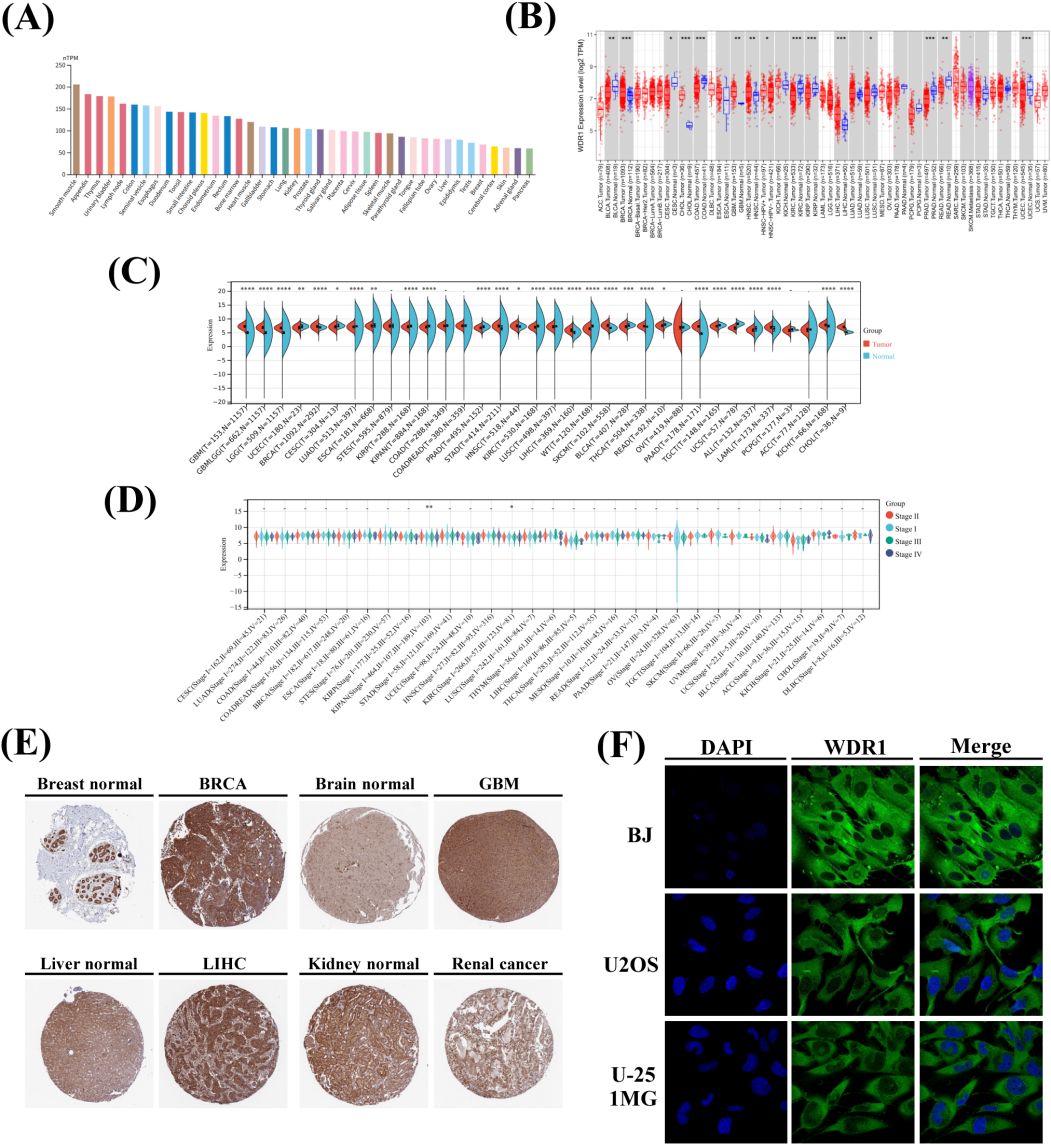
Expression patterns of WDR1 across cancers. **(A)** WDR1 Expression across normal human tissues based on the HPA database. **(B)** Differential expression of WDR1 between tumor and adjacent normal tissues across multiple cancer types in TCGA, analyzed using the Gene_DE module of TIMER2.0. **(C)** Comparison of WDR1 expression between tumor and normal tissues across cancers based on integrated TCGA and GTEx datasets. **(D)** Association between WDR1 expression and pathological stage across different cancer types based on TCGA data. **(E)** Representative immunohistochemical images showing WDR1 protein expression in BRCA, GBM, LIHC and KIRC, along with corresponding normal tissues from the HPA database. **(F)** Representative immunofluorescence images showing the subcellular localization of WDR1 in BJ, U2OS, and U-251 MG cells from the HPA database. Nuclei were stained with DAPI (blue), and WDR1 was shown in green; merged images indicated the overlap of DAPI and WDR1 signals (*P < 0.05; **P < 0.01; ***P < 0.001, ****P < 0.0001, ns, not significant).

We first characterized the baseline expression of WDR1 in normal tissues using the Human Protein Atlas (HPA) database. WDR1 mRNA was broadly expressed across various physiological tissues, with relatively high expression levels observed in smooth muscle, appendix, thymus, urinary bladder, lymph nodes, colon, seminal vesicles, and esophagus (**Figure 1A**).

Next, we assessed the differential expression of WDR1 between tumor and normal tissues using TIMER 2.0. Compared with corresponding normal tissues, WDR1 mRNA expression was significantly upregulated in breast invasive carcinoma (BRCA), cholangiocarcinoma (CHOL), glioblastoma multiforme (GBM), head and neck squamous cell carcinoma (HNSC), and liver hepatocellular carcinoma (LIHC). In contrast, significantly decreased expression was observed in bladder urothelial carcinoma (BLCA), cervical squamous cell carcinoma and endocervical adenocarcinoma (CESC), colon adenocarcinoma (COAD), kidney renal clear cell carcinoma (KIRC), kidney renal papillary cell carcinoma (KIRP), lung squamous cell carcinoma (LUSC), prostate adenocarcinoma (PRAD), rectum adenocarcinoma (READ), and uterine corpus endometrial carcinoma (UCEC) (**Figure 1B**).

Furthermore, normal tissue expression data from the GTEx database were integrated with tumor samples from TCGA. The integrated analysis revealed that WDR1 expression was significantly elevated in 13 cancer types, including GBM, glioma (GBMLGG), brain lower grade glioma (LGG), BRCA, stomach adenocarcinoma (STAD), HNSC, LIHC, skin cutaneous melanoma (SKCM), thyroid carcinoma (THCA), pancreatic adenocarcinoma (PAAD), acute myeloid leukemia (LAML), kidney chromophobe (KICH), and CHOL. In contrast, significantly reduced expression was observed in 15 cancer types, including UCEC, CESC, lung adenocarcinoma (LUAD), esophageal carcinoma (ESCA), KIRP, pan-kidney cohort (KIPAN), PRAD, KIRC, LUSC, Wilms tumor (WT), BLCA, READ, testicular germ cell tumors (TGCT), uterine carcinosarcoma (UCS), and acute lymphoblastic leukemia (ALL) (**Figure 1C**). We next explored the relationship between WDR1 expression and tumor clinical stage. Significant stage-associated differences were observed in KIPAN and KIRC, where WDR1 expression was significantly lower in advanced clinical stages (Stage III and IV) than in early stages (Stage I and II) (**Figure 1D**).

Immunohistochemistry (IHC) data from the HPA database were analyzed to investigate WDR1 protein expression across human cancers. WDR1 expression was increased in BRCA, GBM and LIHC tissues compared with their corresponding normal tissues (**Figure 1E**). Conversely, WDR1 expression was decreased in renal cancer, which was further confirmed by CPTAC proteomic data (**Supplementary Figure 1**). In addition, immunofluorescence-based subcellular localization analysis showed that WDR1 was predominantly localized in the cytoplasm of BJ, U2OS, and U251-MG cells (**Figure 1F**).

### Prognostic and diagnostic value of WDR1

**Figure 2:**
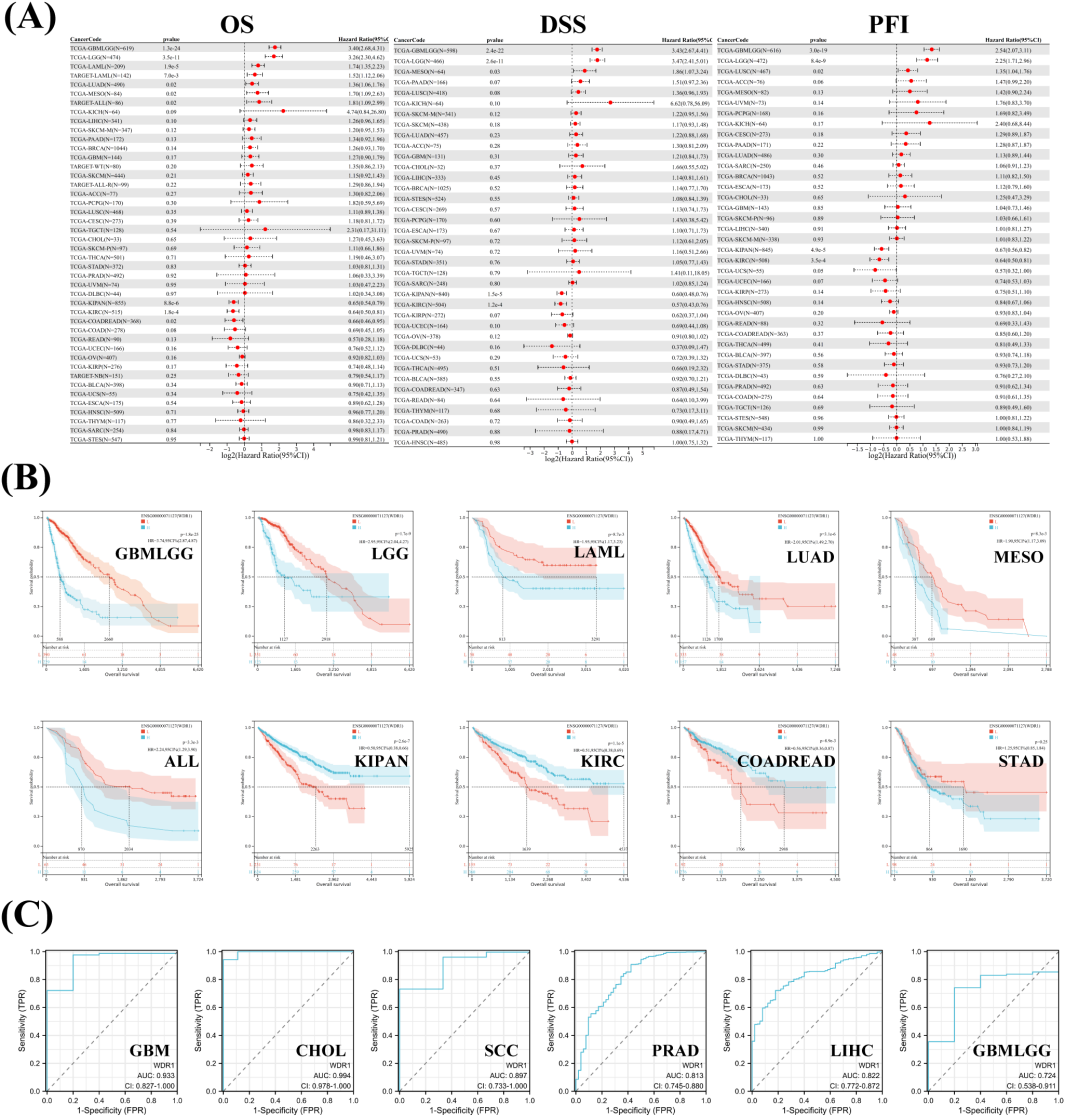
Prognostic and diagnostic values of WDR1. **(A)** Forest plots of univariate Cox regression analyses of WDR1 across cancers for overall survival (OS), disease-specific survival (DSS), and progression-free interval (PFI). Hazard ratios (HRs) and 95% confidence intervals (CIs) were presented for each cancer type. **(B)** Kaplan–Meier survival curves of WDR1 across selected cancers for overall survival (OS). Patients were divided into high- and low-expression groups based on the median WDR1 expression level. **(C)** Receiver operating characteristic (ROC) curves evaluating the diagnostic value of WDR1 in selected cancers. The area under the curve (AUC) was indicated in each panel.

Overall survival (OS), disease-specific survival (DSS), and progression-free interval (PFI) were used to evaluate the prognostic significance of WDR1 expression. The results showed that elevated WDR1 expression was significantly associated with shorter OS in GBMLGG, LGG, LAML, LUAD, mesothelioma (MESO), and ALL. In STAD, increased WDR1 expression exhibited a non-significant trend toward poorer OS. In contrast, higher WDR1 expression correlated with prolonged OS in KIPAN, KIRC, and colon and rectum adenocarcinoma (COADREAD) (**Figures 2A, B**). Consistent with the OS analysis, elevated WDR1 expression was significantly correlated with shorter DSS in GBMLGG, LGG, and MESO, whereas longer DSS was observed in KIPAN and KIRC. Similarly, higher WDR1 expression was associated with shorter PFI in GBMLGG, LGG, and LUSC, but with prolonged PFI in KIPAN and KIRC (**Figure 2A**). Overall, these findings indicate that the prognostic impact of WDR1 varies across cancer types.

To further assess the diagnostic potential of WDR1, receiver operating characteristic (ROC) curves were constructed and the area under the curve (AUC) was calculated across multiple cancer types. WDR1 demonstrated varying diagnostic performance across malignancies. Notably, relatively high diagnostic accuracy (AUC ≥ 0.8) was observed in CHOL (AUC = 0.994, CI: 0.978–1.000), GBM (AUC = 0.933, CI: 0.827–1.000), CESC (AUC = 0.897, CI: 0.733–1.000), LIHC (AUC = 0.822, CI: 0.772–0.872), and PRAD (AUC = 0.813, CI: 0.745–0.880), suggesting that WDR1 may have potential diagnostic value in these cancer types (**Figure 2C**). Moderate diagnostic value was observed in several cancer types, including GBMLGG, COAD, COADREAD, BLCA, and ESCA. In contrast, WDR1 displayed limited to moderate diagnostic performance in KIRC, STAD, BRCA, HNSC, UCEC, KIRP, and LUSC. Relatively low or uncertain diagnostic value was observed in KICH and THCA (**Supplementary Figure 2**).

### Association of WDR1 with DNA methylation and RNA modification regulators

**Figure 3:**
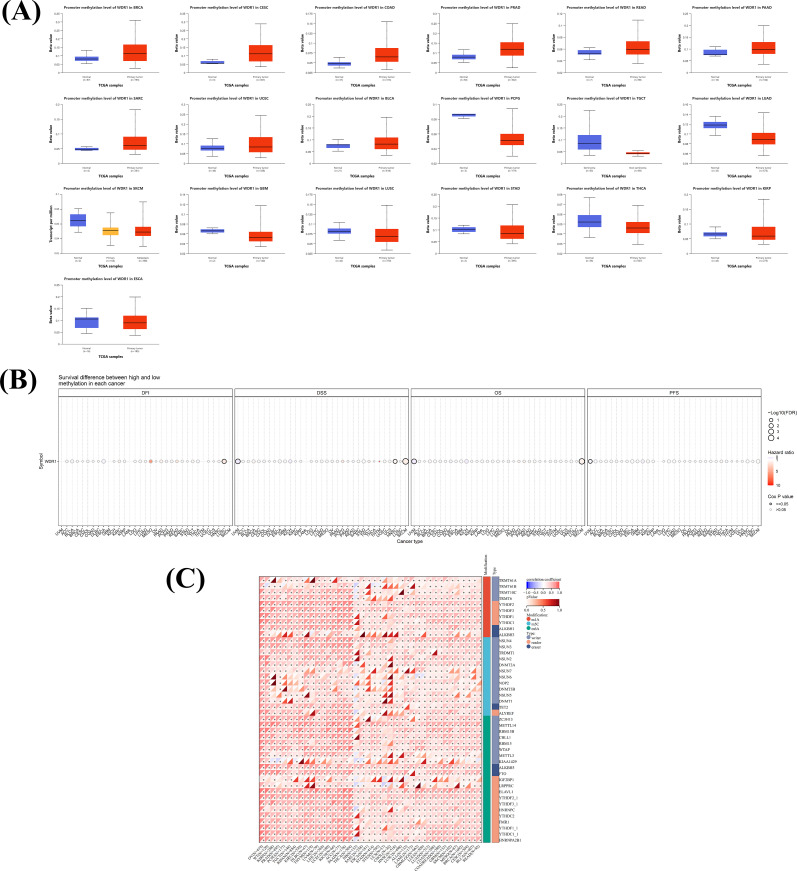
Association of WDR1 with DNA methylation and RNA modification regulators. **(A)** Promoter methylation levels of WDR1 in tumor and normal tissues across selected cancer types based on the UALCAN database. **(B)** Prognostic significance of WDR1 methylation across cancers in the GSCALite platform, including disease-free interval (DFI), disease-specific survival (DSS), overall survival (OS), and progression-free survival (PFS). **(C)** Pearson correlations between WDR1 expression and 44 RNA methylation-related genes, including m1A-, m5C-, and m6A-related regulators, across cancers based on the UCSC database. Only tumor samples were included in this analysis. Color intensity represented the strength and sign of the correlations.

To explore the epigenetic features of WDR1, we systematically evaluated its promoter methylation status across various malignancies using the UALCAN and GSCALite. Compared with normal tissues, WDR1 promoter methylation levels were significantly increased in several cancer types, including BRCA, CESC, COAD, PRAD, READ, PAAD, Sarcoma (SARC), UCEC, and BLCA. In contrast, significantly decreased methylation levels were observed in Pheochromocytoma and Paraganglioma (PCPG), TGCT, LUAD, SKCM, GBM, LUSC, STAD, THCA, KIRP, and ESCA (**Figure 3A**). We further examined the association between WDR1 methylation levels and patient survival using the GSCALite. In uveal melanoma (UVM), higher WDR1 methylation levels were significantly associated with more favorable survival outcomes. In contrast, in LUSC, HNSC, and SKCM, increased methylation levels were correlated with poorer prognosis (**Figure 3B**). These results indicate that the relationship between WDR1 methylation and clinical outcomes varies across cancer types.

We next analyzed the relationship between WDR1 expression and regulators involved in RNA methylation modifications. WDR1 expression showed strong positive correlations with multiple regulators of m1A, m5C, and m6A RNA methylation in most cancer types, with the exception of Neuroblastoma (NB) (**Figure 3C**). These findings suggested that WDR1 expression was closely associated with RNA methylation regulatory networks, although the underlying mechanisms require further investigation.

### Genetic alterations of WDR1 across cancers

**Figure 4:**
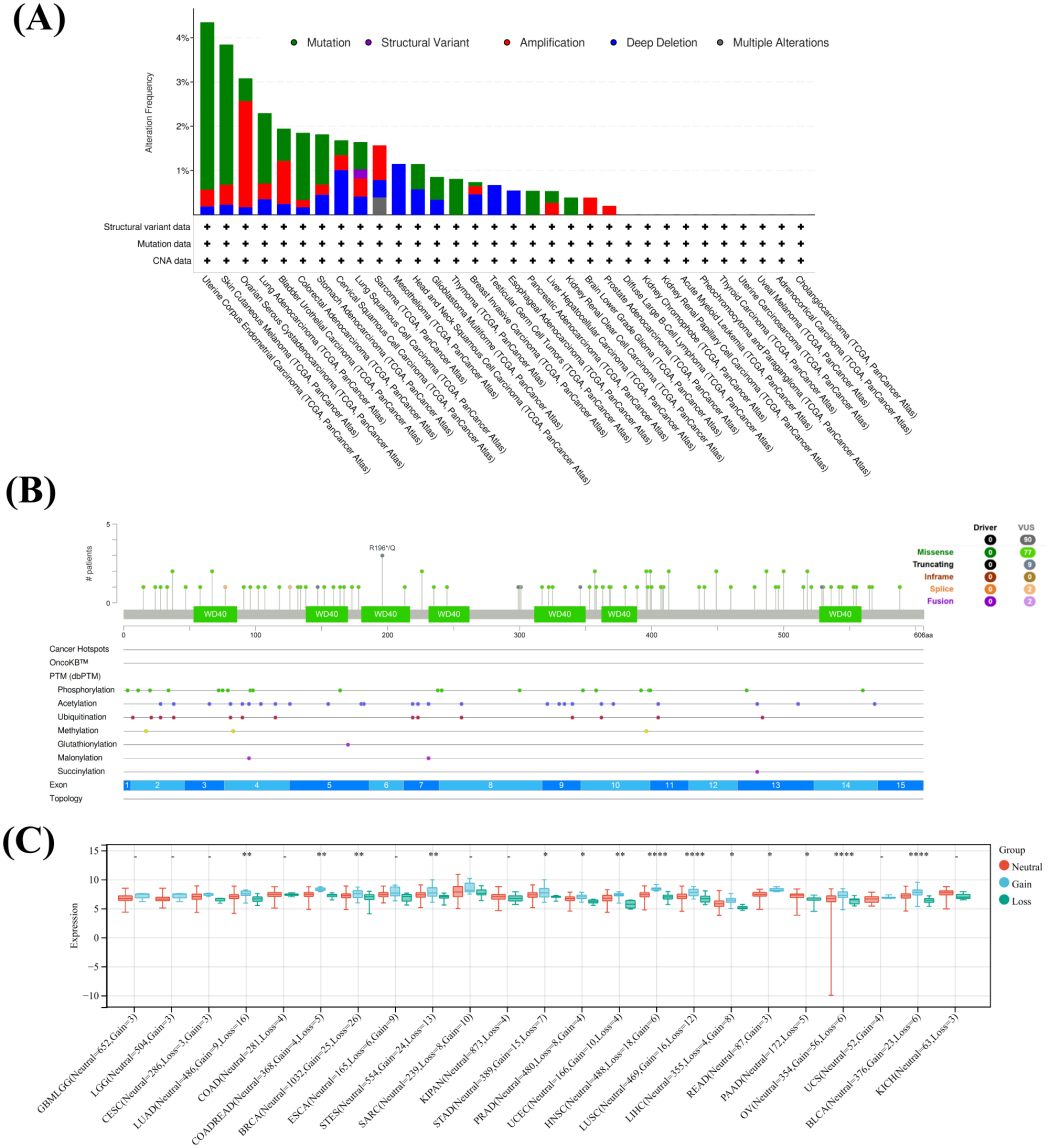
Genetic alterations of WDR1 across cancers. **(A)** Frequency and types of WDR1 genetic alterations across TCGA Pan-Cancer Atlas studies in the cBioPortal database. **(B)** Distribution of mutation sites and mutation types of WDR1 along the protein sequence, illustrated as a lollipop plot from the cBioPortal database. **(C)** Associations between WDR1 copy number variation (CNV) status and its expression across cancer types. Samples were classified into gain, neutral, and loss groups based on GISTIC-derived CNV status. Differences were evaluated using the Kruskal–Wallis test ((*P < 0.05; **P < 0.01, and ****P < 0.0001).

To investigate the genetic alterations associated with WDR1 across cancers, we characterized its genomic alteration profiles in more than 30 tumor types using the cBioPortal database. As shown in **Figure 4A**, UCEC exhibited the highest alteration frequency at 4.35%, predominantly consisting of somatic mutations. This was followed by SKCM (3.85%), Ovarian Serous Cystadenocarcinoma (OV) (3.08%), LUAD (2.3%), and BLCA (1.95%). Among the different types of genetic alterations, missense mutations were the most common, whereas structural variants and multiple alterations occurred less frequently. Notably, gene amplification was the predominant alteration type in OV, with a frequency of 2.4%. Further analysis of mutation distribution showed that most alterations were missense and truncating mutations, which were primarily located within the WD40 domains of WDR1 (**Figure 4B**).

Given that copy number variations (CNVs) are often associated with gene expression changes in cancer, we next evaluated the relationship between WDR1 expression and CNV status across 23 tumor types. Significant associations between WDR1 expression and copy number status were observed in 14 cancers. Specifically, in LUAD, COADREAD, BRCA, Stomach and Esophageal carcinoma (STES), STAD, PRAD, UCEC, HNSC, LUSC, LIHC, READ, OV, BLCA, and PAAD, WDR1 expression levels were higher in samples with copy number gains and lower in those with copy number losses (**Figure 4C**).

### Association of WDR1 with immune cell infiltration

**Figure 5:**
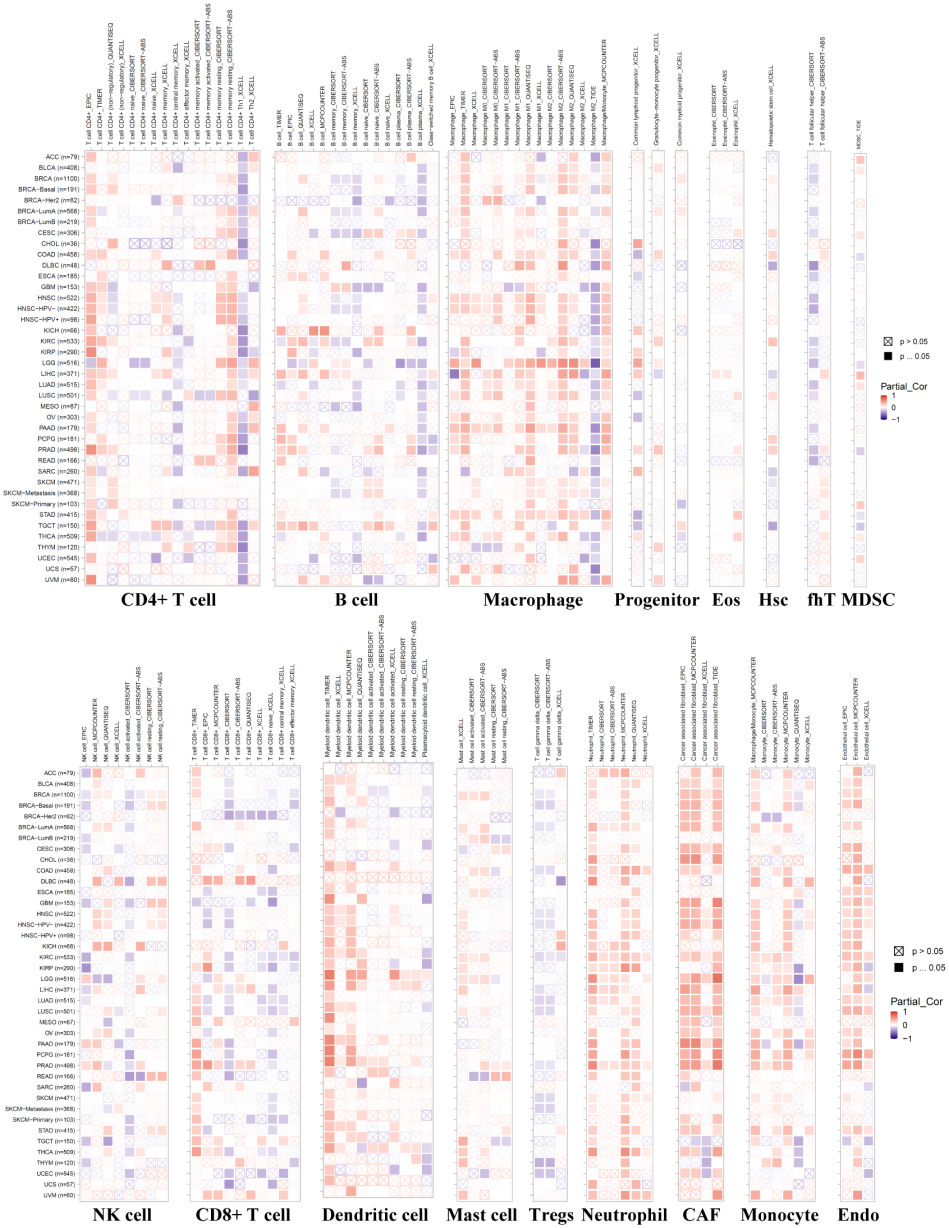
Association between WDR1 expression and immune cell infiltration. Associations between WDR1 expression and infiltration levels of multiple immune cell subsets across cancers, estimated using TIMER2.0 based on TCGA data.

To explore the potential relationship between WDR1 expression and tumor-infiltrating immune cell populations, we performed a systematic analysis using TIMER 2.0 with multiple deconvolution algorithms. As shown in **Figure 5**, WDR1 expression was positively correlated with the infiltration levels of multiple myeloid-derived and stromal cell subsets across the majority of TCGA cancers, particularly macrophages, dendritic cells, neutrophils, cancer-associated fibroblasts (CAFs), monocytes, and endothelial cells (Endo). In contrast, no consistent or significant correlations were observed between WDR1 expression and T cell subsets, B cells, progenitor cells, eosinophils (Eos), hematopoietic stem cells (HSCs), follicular helper T cells (fhTs), myeloid-derived suppressor cells (MDSCs), or natural killer (NK) cells in most cases. Notably, WDR1 expression showed a negative correlation with the infiltration of Tregs in nearly half of the analyzed malignancies, with this trend being particularly evident in KIRC.

Further tumor-specific analyses revealed distinct immune infiltration patterns. WDR1 expression was positively associated with B cell infiltration in KICH, LIHC and TGCT. In Lymphoid Neoplasm Diffuse Large B-cell Lymphoma (DLBC), MESO, TGCT, and UVM, WDR1 expression showed a positive correlation with CD8^+^ T-cell infiltration. Additionally, a positive link between WDR1 and mast cell infiltration was observed in BRCA, KIRC, and PRAD. Conversely, WDR1 expression was negatively correlated with B-cell infiltration in LUAD and CD8^+^ T-cell infiltration in HER2-positive BRCA, ESCA, GBM, and UCEC. Overall, WDR1 expression exhibited more consistent association with myeloid and stromal cell infiltration, whereas its relationship with lymphoid immune cells appears heterogeneous and context-dependent, suggesting a complex and tumor-specific immune landscape.

### Association of WDR1 with immune checkpoint molecules and immune modulators

**Figure 6:**
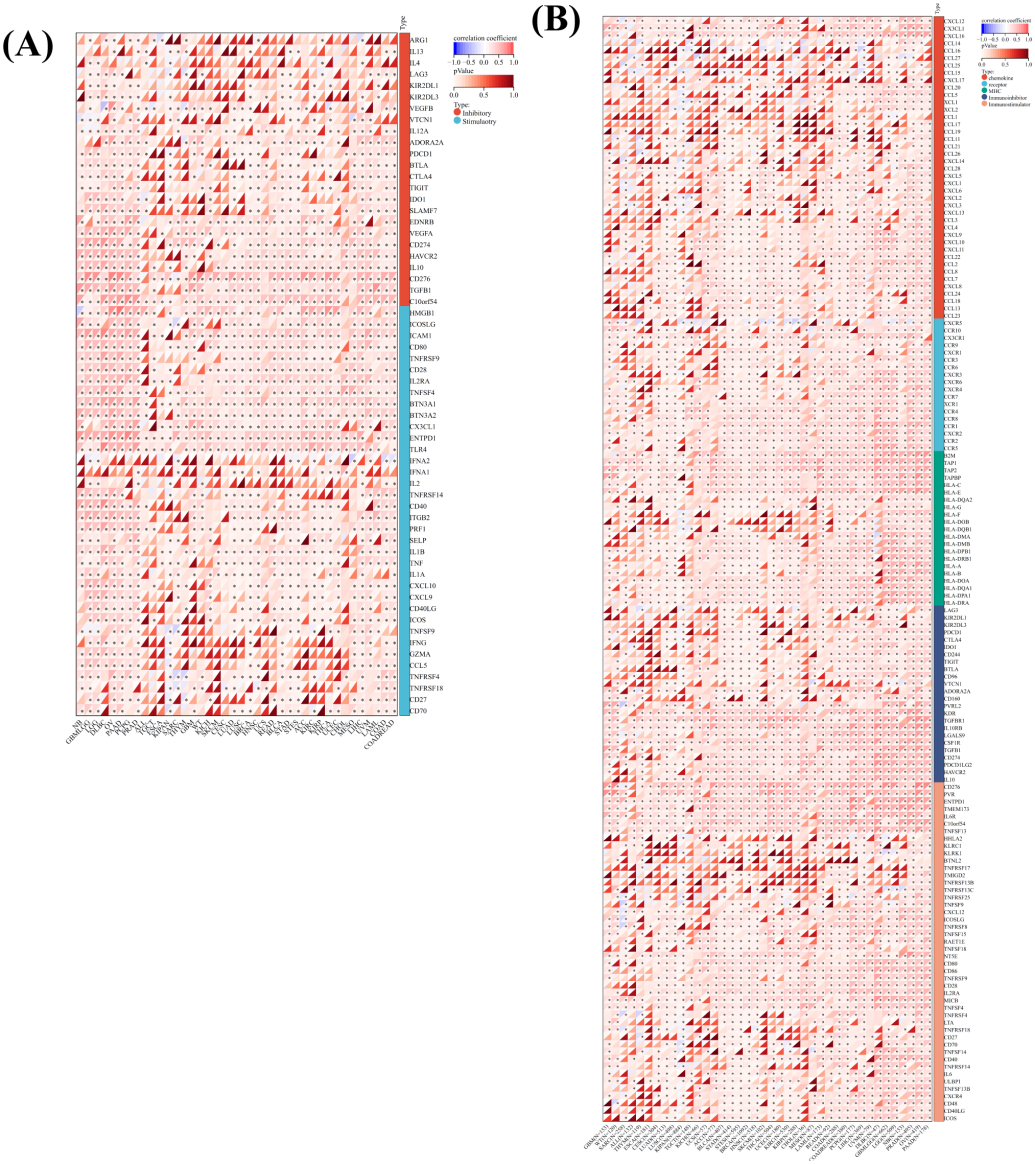
Associations of WDR1 with immune checkpoint molecules and immune modulators. **(A)** Correlations between WDR1 expression and immune checkpoint molecules across cancers based on TCGA data. **(B)** Correlations between WDR1 expression and immune-related genes across five functional categories. Correlations were calculated using Spearman’s rank correlation analysis.

To further investigate the immunological relevance of WDR1, we analyzed the correlations between WDR1 expression and immune checkpoint molecules as well as immune modulators across 40 cancer types. WDR1 expression was broadly and positively correlated with a wide range of immune-related molecules in most cancer types, with the exception of ALL, TGCT, ESCA, KIPAN, SARC, THYM, SKCM, and CHOL. Specifically, WDR1 showed significant positive associations with multiple classical inhibitory checkpoints, including CTLA4, EDNRB, VEGFA, CD274 (PD-L1), HAVCR2 (TIM-3), IL10, CD276, TGFB1, and C10orf54 (VISTA). Simultaneously, WDR1 was also positively correlated with several co-stimulatory and regulatory molecules, such as HMGB1, ICOSLG, ICAM1, CD80, TNFRSF9 (4-1BB), CD28, IL2RA, TNFSF4, BTN3A1, BTN3A2, CX3CL1, ENTPD1, and TLR4 (**Figure 6A**). These findings indicated that WDR1 expression was associated with a tumor microenvironment characterized by the coexistence of immune activation signals and immunoregulatory or inhibitory pathways.

We next examined the associations between WDR1 expression and five categories of immune regulatory molecules, including chemokines, chemokine receptors, major histocompatibility complex (MHC) molecules, immunoinhibitory genes, and immunostimulatory genes. As shown in **Figure 6B**, WDR1 was positively correlated with the majority of the 60 immune modulators analyzed across the 40 cancer types. In terms of chemokines and their receptors, WDR1 was positively associated with factors involved in T cell and Th1-type recruitment (e.g., CXCL16, CXCL10, CXCL11, CX3CL1 and their receptors CXCR3, CXCR6, CX3CR1), as well as factors related to monocyte and myeloid cell recruitment (e.g., CCL2, CCL7, CCL8, CCL18, CXCL8 and their receptors CCR2, CXCR1, CXCR2, CCR1). In addition, WDR1 was positively correlated with chemokines associated with Treg/Th2 cell recruitment (e.g., CCL22, CCL13, CCL24 and receptors CCR4, CCR8, CCR6), as well as molecules involved in immune cell localization and lymphoid structure organization (e.g., CXCL12 and its receptor CXCR4).

Furthermore, WDR1 was positively correlated with multiple genes involved in antigen processing and presentation pathways, including MHC class I and class II–related molecules such as B2M, TAP1, TAP2, TAPBP, HLA-C, HLA-E, HLA-DMB, HLA-DRB1, HLA-DQA1, HLA-DPA1, and HLA-DRA. In addition, WDR1 showed positive associations with both immunoinhibitory genes (e.g., KDR, TGFBR1, IL10RB, TGFB1, CD274, PDCD1LG2, HAVCR2, and IL10) and immunostimulatory genes (e.g., CD276, PVR, ENTPD1, TMEM173, IL6R, C10orf54, CD80, CD86, TNFRSF9, and CD28). Collectively, these results suggested that WDR1 expression was broadly associated with multiple components of the tumor immune microenvironment, including immune cell recruitment, antigen presentation, and immune regulatory pathways, reflecting a complex and context-dependent immune landscape.

### Single-cell expression in the tumor microenvironment and functional states of WDR1

**Figure 7:**
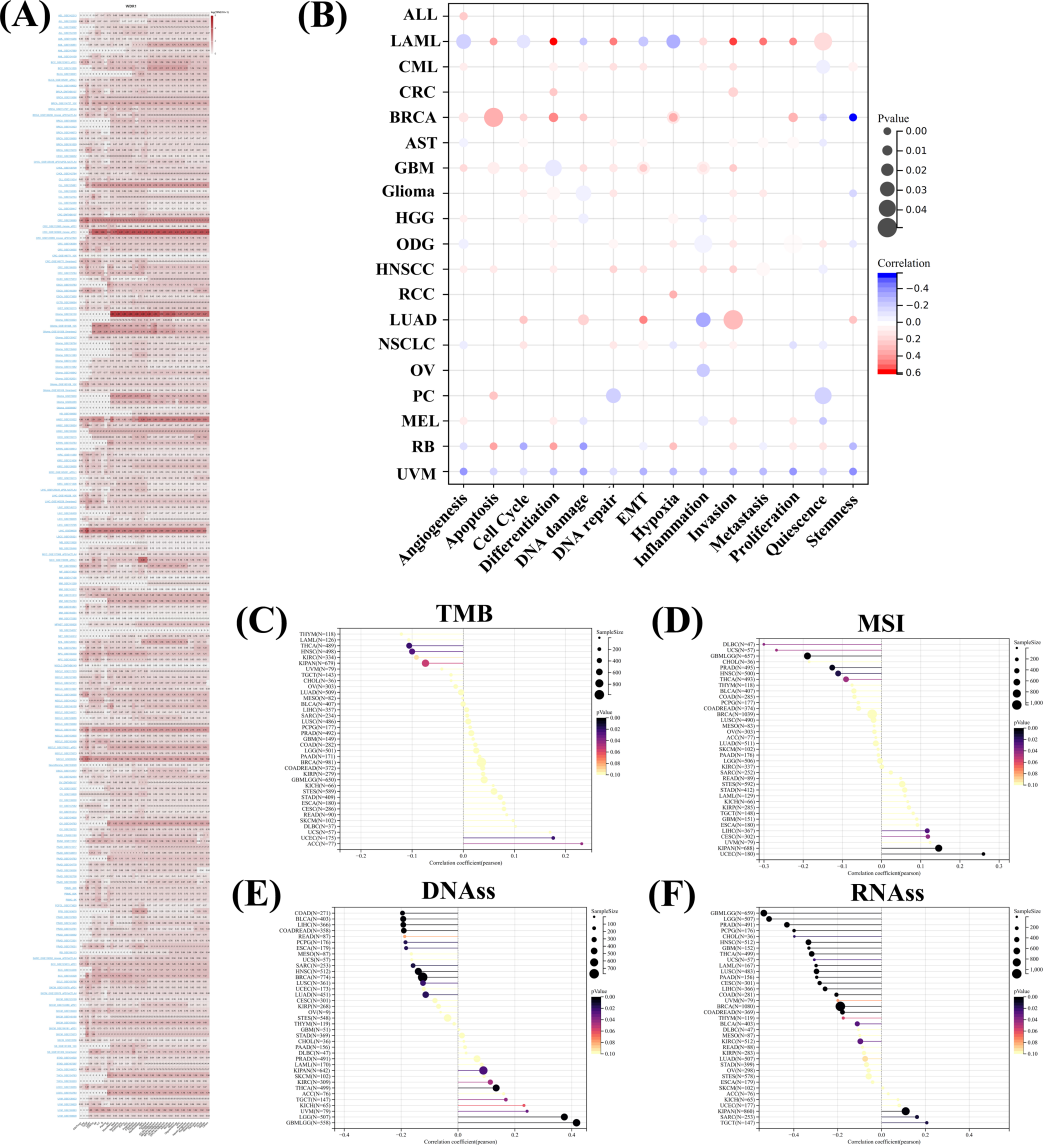
Single-cell expression in the tumor microenvironment and functional states of WDR1. **(A)** Single-cell expression patterns of WDR1 across tumor microenvironment cell populations in pan-cancer datasets from the TISCH database. **(B)** Correlations between WDR1 expression and multiple functional states at the single-cell level across cancer types based on CancerSEA data. Bubble size represented statistical significance, and color indicated the correlation coefficient. **(C–F)** Correlations between WDR1 expression and TMB, MSI, DNAss, and RNAss across cancers, respectively.

To characterize the cellular distribution of WDR1 within the tumor microenvironment (TME), we performed a systematic analysis using the TISCH database. The results showed that WDR1 expression exhibited marked tumor-type specificity and cellular heterogeneity. As shown in **Figure 7A**, in several malignancies, including gastrointestinal stromal tumor (GIST), LUSC, Merkel cell carcinoma (MCC), mycosis fungoides (MF), nasopharyngeal carcinoma (NPC), osteosarcoma (OS), primary cutaneous follicle center lymphoma (PCFCL), SARC, and small cell lung cancer (SCLC), WDR1 expression was broadly distributed across multiple immune and stromal cell populations. In contrast, more cell-type–restricted expression patterns were observed in other tumor types. For example, in HNSC, WDR1 expression was relatively enriched in exhausted CD8^+^ T cells (CD8^+^ Tex), innate lymphoid cells (ILCs), and NK cells. Consistently, elevated WDR1 expression was also observed in ILCs and NK cells in peripheral blood mononuclear cell (PBMC) datasets (30K, 60K, and 8K). In addition, in non-Hodgkin lymphoma (NHL) datasets (GSE128531 and GSE147944), WDR1 expression was enriched in cell populations annotated as acinar, ductal, pericytes, and stellate cells. These findings collectively suggested that WDR1 expression displayed substantial heterogeneity across both tumor types and cellular compartments within the TME.

To further explore the functional associations of WDR1 at the single-cell level, we analyzed its correlations with 14 functional states using the CancerSEA database. As shown in **Figure 7B**, WDR1 exhibited pronounced cancer-type–specific functional patterns. Among the 19 cancer types analyzed, the most extensive associations were observed in LAML, BRCA, LUAD, and UVM. In LAML, WDR1 was positively correlated with multiple biological processes, including apoptosis, differentiation, DNA repair, inflammation, invasion, metastasis, and quiescence. In BRCA, WDR1 showed positive correlations with angiogenesis, apoptosis, cell cycle, differentiation, DNA damage, hypoxia, and proliferation, with the strongest association observed for differentiation. Similarly, in LUAD, WDR1 was positively correlated with cell cycle, DNA damage, epithelial–mesenchymal transition (EMT), and invasion. In contrast, WDR1 displayed predominantly negative correlations with functional states in UVM, including angiogenesis, differentiation, proliferation, and stemness. Additional tumor-specific negative associations were also identified, such as negative correlations with DNA repair and quiescence in pancreatic cancer (PC) and with inflammation-related processes in OV. Overall, these findings suggested that WDR1 was associated with diverse functional states at the single-cell level, with substantial variability across tumor types.

### Association of WDR1 with genomic instability and tumor stemness

Building on the observed associations between WDR1 expression and immune-related features, we next explored its potential relationships with intrinsic tumor characteristics, including genomic instability and cancer stemness. We first performed correlation analyses between WDR1 expression and two commonly used indicators of genomic instability, tumor mutational burden (TMB) and microsatellite instability (MSI). WDR1 expression was significantly negatively correlated with TMB in several cancer types, including THCA, HNSC, and KIPAN, while positive correlations were observed in adrenocortical carcinoma (ACC) and UCEC (**Figure 7C**). Regarding MSI, WDR1 expression showed significant negative correlations in DLBC, UCS, GBMLGG, PRAD, HNSC, and THCA, whereas positive correlations were identified in UCEC, KIPAN, CESC, and LIHC (**Figure 7D**).

To assess the association between WDR1 expression and tumor stemness, we analyzed its correlation with stemness indices based on DNA methylation (DNAss) and mRNA expression (RNAss) across 33 cancer types. WDR1 expression was negatively correlated with DNAss in multiple cancers, including COAD, BLCA, LIHC, COADREAD, PCPG, ESCA, SARC, HNSC, BRCA, LUSC, and LUAD, while positive correlations were observed in GBMLGG, LGG, UVM, TGCT, THCA, KIRC, and KIPAN (**Figure 7E**). Similarly, WDR1 expression was negatively correlated with RNAss in the majority of cancer types, including GBMLGG, LGG, PRAD, PCPG, and THCA, whereas positive correlations were observed in a limited number of cancers, such as TGCT, SARC, and KIPAN (**Figure 7F**). These findings suggested that the associations between WDR1 expression, genomic instability, and tumor stemness were heterogeneous and vary across cancer types.

### Molecular interaction network and functional enrichment of WDR1

**Figure 8:**
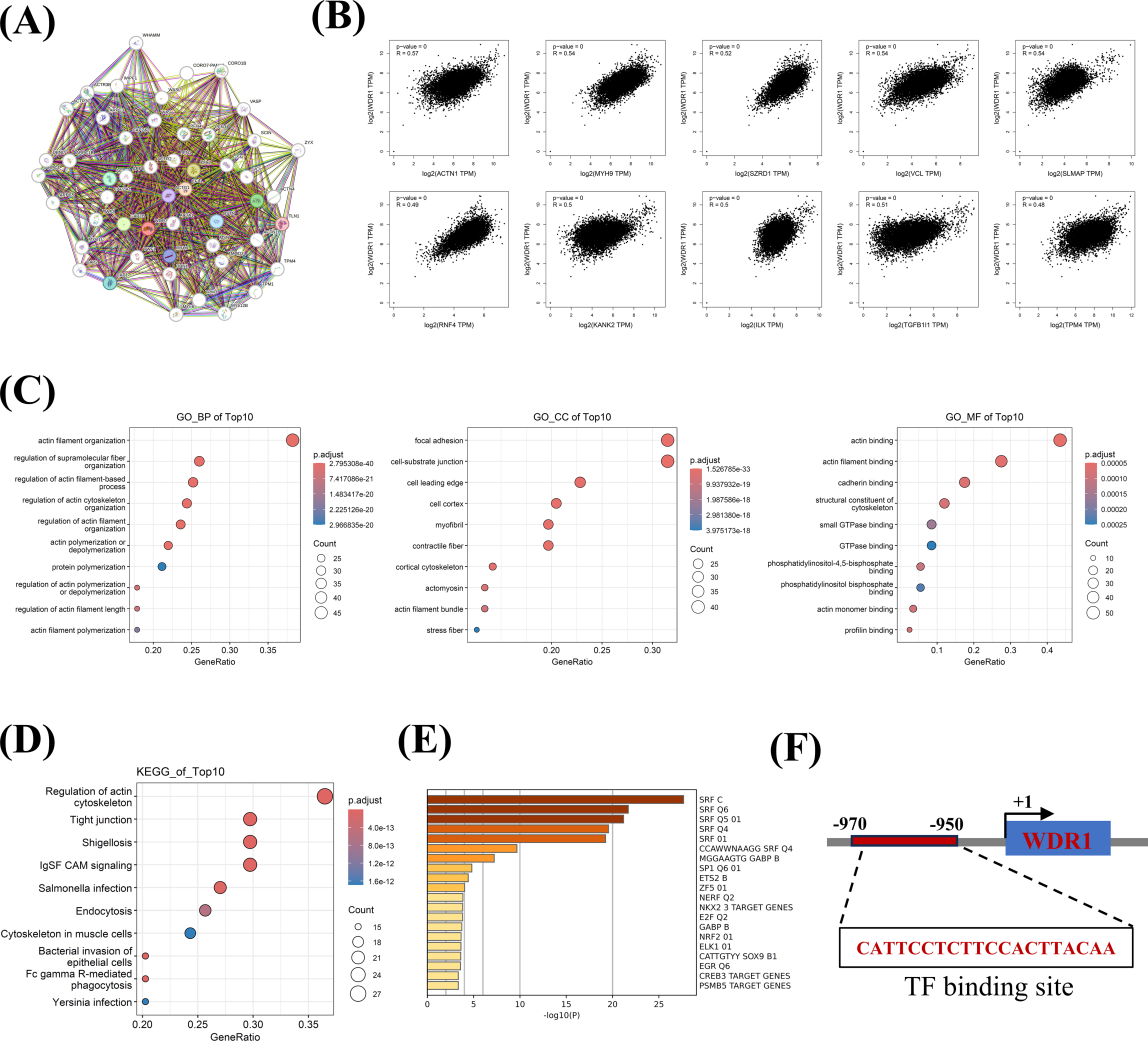
Molecular interaction network and functional enrichment of WDR1. **(A)** A total of 50 WDR1-interacting proteins were identified by using the STRING database. **(B)** Correlations between WDR1 and the top 10 positively correlated genes from the integrated gene set derived from STRING and GEPIA2. **(C)** GO enrichment analysis of WDR1-related genes in biological process (BP), cellular component (CC), and molecular function (MF) categories. **(D)** KEGG pathway enrichment analysis of WDR1-related genes. **(E)** Transcription factor enrichment analysis of WDR1-related genes. **(F)** Predicted transcription factor binding site in the WDR1 promoter region based on JASPAR analysis.

To further explore the potential biological functions associated with WDR1, we constructed a protein–protein interaction (PPI) network using the STRING database with a confidence score > 0.7, identifying 50 WDR1-interacting proteins (**Figure 8A**). In parallel, based on TCGA pan-cancer expression data integrated in the GEPIA2 platform, we identified the top 100 genes most significantly correlated with WDR1 expression. Among these genes, the top 10 positively correlated genes included ACTN1 (R = 0.57), MYH9 (R = 0.54), SZRD1 (R = 0.52), VCL (R = 0.54), SLMAP (R = 0.54), RNF4 (R = 0.49), KANK2 (R = 0.50), ILK (R = 0.50), TGFB1I1 (R = 0.51), and TPM4 (R = 0.48) (**Figure 8B**).

The integrated gene set, consisting of WDR1-interacting proteins and WDR1-correlated genes, was defined as WDR1-related genes and subsequently subjected to Gene Ontology (GO) and Kyoto Encyclopedia of Genes and Genomes (KEGG) pathway enrichment analyses. GO analysis revealed significant enrichment in biological processes related to actin filament organization, regulation of supramolecular fiber organization, regulation of actin filament-based processes, regulation of actin cytoskeleton organization, regulation of actin filament organization, actin polymerization or depolymerization, and protein polymerization. In terms of cellular components, enriched terms included focal adhesion, cell–substrate junction, cell leading edge, cell cortex, myofibril, and contractile fiber. Molecular function analysis further demonstrated enrichment in actin binding, actin filament binding, cadherin binding, and structural constituents of the cytoskeleton (**Figure 8C**). Consistently, KEGG pathway enrichment analysis identified several significantly enriched pathways, including regulation of the actin cytoskeleton, tight junction, Shigellosis, IgSF CAM signaling, Salmonella infection, Endocytosis, and Cytoskeleton in muscle cells (**Figure 8D**). Taken together, these results indicated that WDR1-associated genes were strongly enriched in pathways related to cytoskeletal organization and cell structural dynamics.

To investigate potential upstream regulatory features, we performed transcription factor (TF) enrichment analysis using the integrated gene set. SRF-related transcription factors (including SRF C, SRF Q6, SRF Q5, and SRF Q4) showed the most significant enrichment, followed by GABP, SP1, E2F, NRF2, and ELK1 (**Figure 8E**). Using the JASPAR database, we further identified a potential SRF binding motif within the WDR1 promoter region. A high-scoring motif was detected between −970 and −950 bp relative to the transcription start site (TSS), suggesting a potential role of SRF in the transcriptional regulation of WDR1 (**Figure 8F**).

### GSEA enrichment and drug sensitivity associated with WDR1

**Figure 9:**
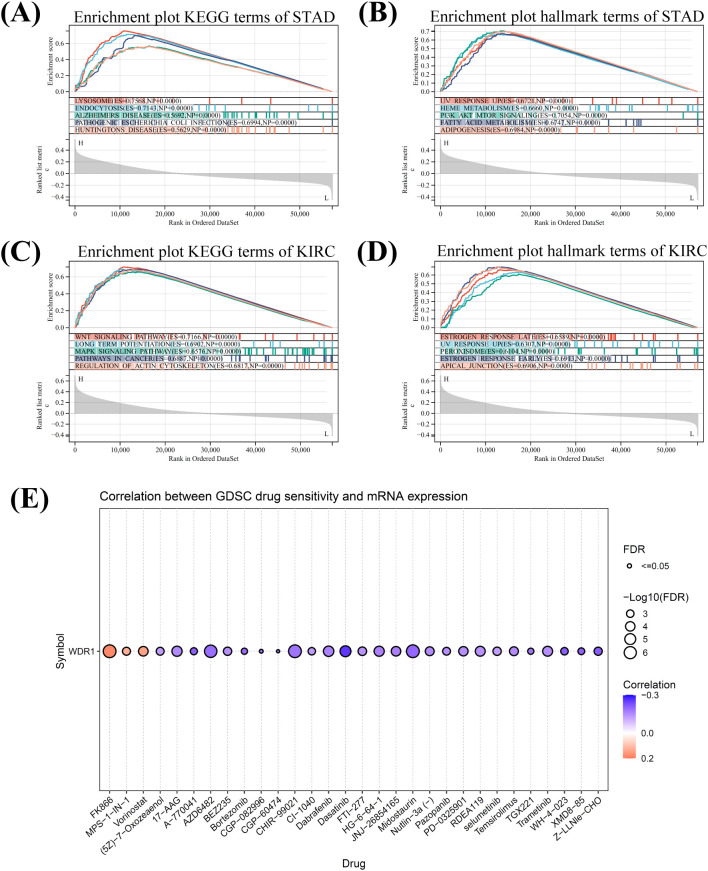
GSEA enrichment and drug sensitivity associated with WDR1. **(A)** KEGG enrichment in STAD. **(B)** Hallmark enrichment terms in STAD. **(C)** KEGG enrichment in KIRC. **(D)** Hallmark enrichment terms in KIRC. **(E)** Associations between WDR1 expression and drug sensitivity based on GDSC data from GSCALite. Drug response was evaluated using half-maximal inhibitory concentration (IC50) value. FDR < 0.05 was considered statistically significant.

Our preceding analyses showed that WDR1 was significantly down-regulated in KIRC tumor tissues compared with normal controls (**Figures 1B–D**), and higher WDR1 expression was associated with favorable survival outcomes (**Figures 2A, B**). In contrast, in STAD, WDR1 was up-regulated in tumor tissues (**Figure 1C**) and tended to be associated with poorer prognosis (**Figures 2A, B**). To explore the potential biological processes underlying these contrasting patterns, we performed GSEA in both cohorts. In STAD, WDR1 expression was predominantly associated with KEGG pathways related to lysosome, endocytosis, Alzheimer’s disease, pathogenic Escherichia coli infection, and Huntington’s disease (**Figure 9A**). In addition, HALLMARK enrichment analysis indicated WDR1 expression was associations with UV response, heme metabolism, PI3K/AKT/mTOR signaling, fatty acid metabolism, and adipogenesis (**Figure 9B**). In KIRC, KEGG analysis identified enrichment in the Wnt signaling pathway, long-term potentiation, MAPK signaling pathway, pathways in cancer, and regulation of the actin cytoskeleton (**Figure 9C**), whereas HALLMARK analysis highlighted associations with estrogen response (early and late), UV response up, peroxisome, and apical junction (**Figure 9D**). Collectively, these results suggested that WDR1 was associated with distinct pathway signatures across different tumor contexts.

To assess the association between WDR1 expression and drug response, we analyzed the correlation between WDR1 expression and drug sensitivity using the GDSC database. WDR1 expression was significantly correlated with the half-maximal inhibitory concentration (IC50) values of multiple targeted therapies and chemotherapeutic agents. Among the top 30 correlated drugs, three—FK866, MPS-1-IN-1, and Vorinostat—showed positive correlations with WDR1 expression, indicating reduced sensitivity in WDR1-high cells. In contrast, the remaining 27 compounds, including (5Z)-7-Oxozeaenol, 17-AAG, A-770041, AZD6482, BEZ235, Bortezomib, CGP-082996, CGP-60474, CHIR-99021, CI-1040, Dabrafenib, Dasatinib, FTI-277, HG-6-64-1, JNJ-26854165, Midostaurin, Nutlin-3a (-), Pazopanib, PD-0325901, RDEA 119, selumetinib, Temsirolimus, TGX221, Trametinib, WH-4-023, XMD8-85, and Z-LLNle-CHO, exhibited negative correlations, suggesting increased sensitivity in WDR1-high cells (**Figure 9E**). Overall, these results indicate that WDR1 expression was associated with differential drug responses across multiple therapeutic agents.

### Experimental validation of the context-dependent roles of WDR1 in KIRC and STAD

**Figure 10:**
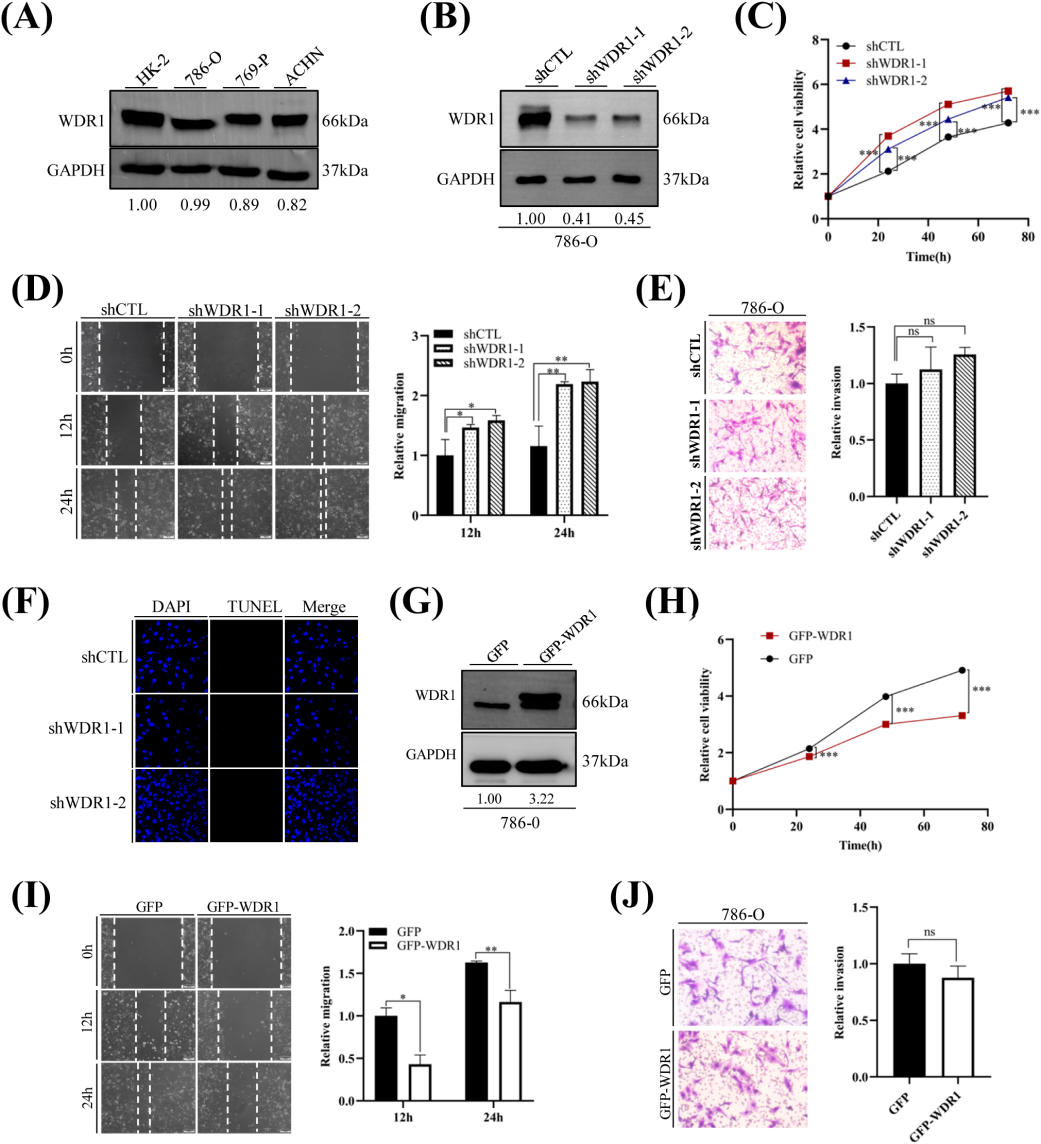
Experimental validation of the context-dependent roles of WDR1 in KIRC. **(A)** Protein expression levels of WDR1 in normal renal epithelial cell line (HK-2) and three renal cancer cell lines. Relative protein levels were quantified and presented below the corresponding bands. **(B)** Western blot analysis of WDR1 knockdown efficiency in 786-O cells. Relative protein levels were presented below the corresponding bands. **(C)** CCK-8 assay in control (shCTL) and WDR1 knockdown (shWDR1) 786-O cells. **(D)** Wound healing assay in shCTL and shWDR1 786-O cells. Images were captured at 0, 12, and 24 hours, and quantitative analysis was shown on the right. **(E)** Transwell invasion assay in shCTL and shWDR1 786-O cells. Representative images and quantitative analysis were shown. **(F)** Apoptosis assay in shCTL and shWDR1 786-O cells. **(G)** Western blot analysis of WDR1 overexpression efficiency in 786-O cells. Relative protein levels were shown below the corresponding bands. **(H)** CCK-8 assay in control (GFP) and WDR1- overexpressing (GFP-WDR1) 786-O cells. **(I)** Wound healing assay in control (GFP) and WDR1-overexpressing (GFP-WDR1) 786-O cells. Images were captured at 0, 12, and 24h, and quantitative analysis was shown on the right. **(J)** Transwell invasion assay in control (GFP) and WDR1-overexpressing (GFP-WDR1) 786-O cells. Representative images and quantitative analysis were shown. Data were presented as mean ± SEM. *P < 0.05, **P < 0.01, and ***P < 0.001.

Our bioinformatic analyses indicated that WDR1 expression was significantly down-regulated in KIRC tumor tissues and that higher WDR1 expression was associated with more favorable clinical outcomes. To further investigate the potential biological role of WDR1 in KIRC, we performed *in vitro* experiments using the 786-O cell line, in which the protein level of WDR1 was shown in **Figure 10A**. Stable WDR1-knockdown cell lines were established through shRNA-mediated lentiviral transduction, followed by antibiotic selection to obtain stably transduced populations. The knockdown efficiency was further confirmed at the protein level prior to functional assays (**Figure 10B**). Functional assays revealed that WDR1 knockdown significantly promoted cell proliferation and migration (**Figures 10C, D**). In addition, WDR1 knockdown resulted in a slight but non-significant increase in cell invasive capacity (**Figure 10E**), which may reflect the distinct biological processes underlying cell migration and invasion, as tumor cell invasion additionally depends on extracellular matrix degradation and cell–matrix interactions. The relatively limited sample size may also have reduced the statistical power to detect subtle differences. Apoptosis assays further showed that WDR1 knockdown did not induce apoptosis (**Figure 10F**). In contrast, WDR1 overexpression in 786-O cells markedly inhibited cell proliferation and migration and was accompanied by a slight, although non-significant, reduction in invasive capacity (**Figures 10G–J**).

**Figure 11:**
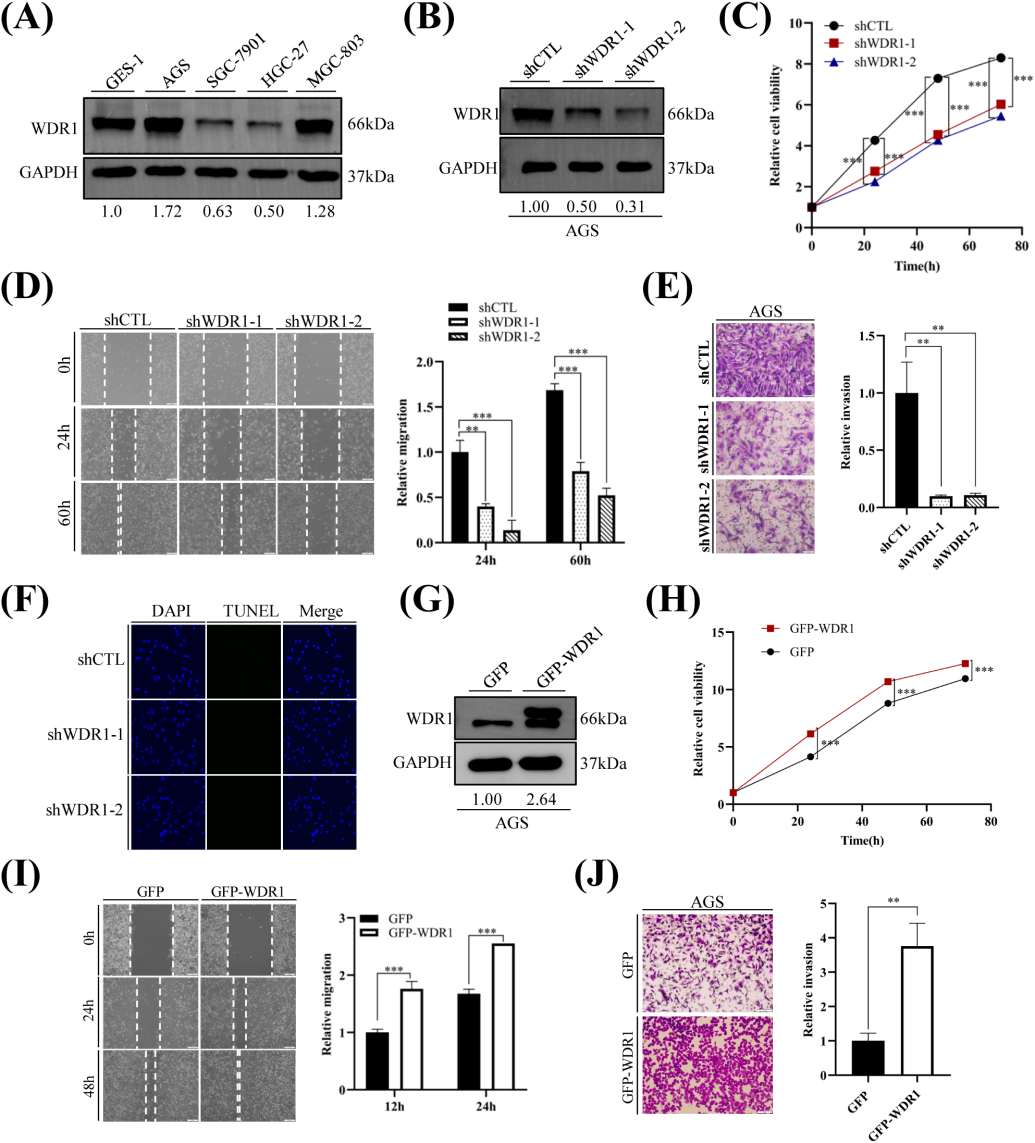
Experimental validation of the context-dependent roles of WDR1 in STAD. **(A)** Protein expression levels of WDR1 in the normal gastric epithelial cell line (GES-1) and four gastric cancer cell lines. Relative protein levels were quantified and presented below the corresponding bands. **(B)** Western blot analysis of WDR1 knockdown efficiency in AGS cells. Relative protein levels were quantified and presented below the corresponding bands. **(C)** CCK-8 assay showed reduced proliferation of AGS cells after WDR1 knockdown. **(D)** Wound healing assay revealed reduced migratory ability of AGS cells after WDR1 knockdown. Images were captured at 0, 24, and 60h. Quantitative analysis of relative migration was shown on the right. **(E)** Transwell invasion assay showed reduced invasive ability of AGS cells after WDR1 knockdown. Representative images and quantitative analysis were shown. **(F)** Apoptosis assay in shCTL and shWDR1 AGS cells. **(G)** Western blot analysis of WDR1 overexpression efficiency in AGS cells. Relative protein levels were quantified and presented below the corresponding bands. **(H)** CCK-8 assay in control (GFP) and WDR1-overexpressing (GFP-WDR1) AGS cells. **(I)** Wound healing assay in control (GFP) and WDR1-overexpressing (GFP-WDR1) AGS cells. Images were captured at 0, 24, and 48h. Quantitative analysis of relative migration was shown on the right. **(J)** Transwell invasion assay in control (GFP) and WDR1-overexpressing (GFP-WDR1) AGS cells. Representative images and quantitative analysis were shown. Data were presented as mean ± SEM. *P < 0.05, **P < 0.01, and ***P < 0.001.

In contrast to KIRC, WDR1 was up-regulated in STAD, and elevated WDR1 expression was associated with poorer prognosis. To further explore the context-dependent role of WDR1, we chose AGS cells to perform *in vitro* experiments, and expression levels of WDR1 were presented in **Figure 11A**. Silencing of WDR1 markedly suppressed the proliferation, migration and invasion abilities of AGS cells, whereas no effect on cell apoptosis was observed (**Figures 11B–F**). Conversely, overexpressed WDR1 enhanced cell proliferation, migration and invasion in AGS cells (**Figures 11G–J**). Collectively, these findings indicate that WDR1 exerts context-specific functions in tumor progression, displaying different regulatory effects on cellular behaviors in KIRC and STAD models.

## Discussion

Pan-cancer analyses provide a valuable framework for uncovering both shared and cancer-type-specific features across malignancies, thereby facilitating cancer prevention and therapeutic target discovery. WDR1, a key regulator of actin cytoskeleton dynamics, is essential for maintaining cellular structural homeostasis and regulating cell migration and immune responses. Increasing evidence has implicated that aberrant WDR1 expression is involved in tumor initiation, progression, and prognosis across multiple cancer types, including NSCLC, breast cancer, and hepatocellular carcinoma (32, 33, 35). In this study, we systematically investigated the role of WDR1 across cancers by integrating multiple public datasets to analyze its expression patterns, prognostic value, epigenetic regulation, genetic alterations, immune cell infiltration, immune checkpoint molecules, functional heterogeneity at the single-cell level, TMB, MSI, tumor stemness, functional enrichment and drug sensitivity. In addition, experimental validation was performed in KIRC and STAD models.

Previous studies have reported that WDR1 was upregulated in multiple cancers, including NSCLC, GBM, BRCA, LIHC, THCA, PRAD, and PDAC, where it contributed to tumor progression. Consistent with these findings, we observed elevated WDR1 expression in GBM, BRCA, LIHC, HNSC, and THCA. In contrast, WDR1 expression was decreased in KIRC, KIRP, BLCA, PRAD, UCEC, READ, and CESC, with some discrepancies observed compared to previous reports (e.g., PRAD) (37). Moreover, discrepancies in WDR1 expression across different datasets, particularly in LUAD and LUSC, may reflect differences in sample composition, data acquisition methods, and technical platforms. Collectively, these findings indicate substantial cancer-type-specific heterogeneity in WDR1 expression. Consistently, survival analyses revealed that elevated WDR1 expression was associated with poorer prognosis in GBMLGG and LGG, but with more favorable outcomes in KIRC and KIPAN, further supporting the context-dependent roles of WDR1 across cancers and highlighting its potential clinical relevance as a prognostic biomarker.

By integrating multi-omics analyses with experimental validation, we demonstrated marked cancer-type specificity of WDR1. Its associations with epigenetic regulation (including DNA methylation and RNA methylation regulators), genetic alterations, genomic instability, cancer stemness, and immune-related features all exhibited distinct, context-dependent patterns. Functional experiments further supported these observations, showing that WDR1 exerted divergent biological effects in KIRC and STAD.

An important finding of this study was the close association between WDR1 and the tumor immune microenvironment. WDR1 expression was positively associated with infiltration of multiple myeloid and stromal cell populations, including macrophages, dendritic cells, neutrophils, and cancer-associated fibroblasts, whereas its associations with lymphocyte populations (T cells, B cells, and NK cells) were relatively weak or exhibited pronounced tumor-type-specific heterogeneity. Notably, WDR1 expression was negatively associated with Treg infiltration in several cancers, particularly KIRC. Given the established role of actin cytoskeleton remodeling in regulating immune cell migration and adaptation within complex tissue environments (47, 48), this finding may be related to the function of WDR1 in cofilin-mediated actin filament disassembly and cytoskeletal remodeling. Consistently, WDR1 expression was positively associated with multiple chemokines and their receptors involved in the recruitment of T cells (e.g., CXCL10/11–CXCR3/6) and myeloid cells (e.g., CCL2, CXCL8–CCR1/2). Interestingly, although WDR1 expression was also positively associated with the Treg-related CCL22–CCR4 axis, Treg infiltration itself showed a negative correlation with WDR1 expression. This discrepancy may reflect the complex regulation of Treg accumulation, which depends not only on chemotactic recruitment but also on expansion, survival, and phenotypic stability within the tumor microenvironment (49). In addition, because the CCL22–CCR4 axis also recruits other CCR4^+^ immune subsets, such as Th2 and Th17 cells, recruitment of these cells may further dilute Treg enrichment (50). These findings suggest that WDR1 may influence immune cell migration and spatial adaptation within the tumor microenvironment, thereby contributing to tumor-type-specific immune landscapes.

Furthermore, WDR1 expression showed broad positive correlations with multiple immune checkpoint molecules (such as PD-L1, CTLA4, VISTA, and TIM-3), together with co-stimulatory molecules (including CD80, CD86, 4-1BB, and CD28) and antigen presentation-related molecules. This pattern suggested an immune-inflamed but regulated tumor microenvironment and was consistent with adaptive immune resistance, in which immune activation induces compensatory up-regulation of immune checkpoint molecules (e.g., PD-L1 and CTLA4) to maintain immune homeostasis (51, 52). These associations likely reflect the overall immune context rather than a direct regulatory role of WDR1 in antigen presentation.

As a key regulator of actin dynamics, WDR1 promotes cofilin-mediated actin filament disassembly and plays critical roles in maintaining cell morphology, regulating cell migration, and supporting immune cell function (10, 14, 26, 53). Consistently, our PPI network and functional enrichment analyses showed that WDR1-associated genes were significantly enriched in pathways related to actin filament organization, cytoskeletal regulation, focal adhesion, and cell–matrix interactions. KEGG analysis further supported its involvement in cytoskeletal remodeling and cell adhesion-related pathways. In addition, transcription factor prediction analyses suggested that SRF may function as an upstream regulator of WDR1. WDR1 expression was positively associated with multiple canonical SRF target genes, including ACTN1, MYH9, and VCL, and potential SRF-binding sites were also identified within the WDR1 promoter region. Since SRF transcriptional activity is closely linked to actin cytoskeleton dynamics through the MRTF–SRF signaling pathway, WDR1 may influence the globular actin (G-actin)/filamentous actin (F-actin) balance and thereby modulate MRTF–SRF signaling axis (54, 55). Together, these findings suggest that WDR1 may participate in coordinating cytoskeletal remodeling, transcriptional regulation and immune-related cellular functions across cancers.

A major finding of this study was the tumor-suppressive role of WDR1 in KIRC. This was supported by its down-regulation in tumor tissues, its positive association with favorable prognosis, and its inhibitory effects on proliferation, migration and invasion in 786-O cells. In contrast to the pro-tumorigenic roles reported in most other cancer types, these results highlight a strong context-dependent function of WDR1. This discrepancy may be closely related to the unique metabolic reprogramming, cytoskeletal dynamics, and immune microenvironment remodeling characteristic of KIRC (56). Notably, WDR1 expression was positively correlated with the infiltration of myeloid cells and antigen-presenting cells, but negatively correlated with Treg infiltration, suggesting that WDR1 may be associated with a relatively immune-activated microenvironment in KIRC. Together, these findings indicate that WDR1 may exert tumor-suppressive effects in KIRC through coordinated regulation of cytoskeletal homeostasis and tumor immune microenvironment remodeling. Nevertheless, several limitations of this study should be acknowledged. Although functional validation was performed in both KIRC and STAD models using stable WDR1 overexpression and knockdown systems, only one representative cell line was included for each cancer type. Further validation in additional cell lines and experimental models would help strengthen the generalizability of these findings. In addition, the associations between WDR1 and immune regulation, cytoskeletal remodeling, and related signaling pathways were mainly inferred from integrated bioinformatic analyses, and the underlying molecular mechanisms warrant further investigation through *in vitro* and *in vivo* studies.

In conclusion, this study systematically characterized the associations of WDR1 expression with multiple molecular and clinical features across cancers, providing a comprehensive overview of its context-dependent roles. These findings provide mechanistic insights into the interplay between cytoskeletal regulation and tumor progression, and further suggest that WDR1 may serve as a promising clinical biomarker and therapeutic target, particularly in KIRC and STAD.

## Data Availability

The data that support the findings of this study are openly available in public repositories. Transcriptomic and clinical data across cancer types were obtained from The Cancer Genome Atlas (TCGA) and Genotype-Tissue Expression (GTEx) databases through the UCSC Xena platform at https://xenabrowser.net/datapages/. Proteomic validation data for the CPTAC KIRC cohort were obtained from the CPTAC/PDC database under accession number PDC000127 at https://proteomic.datacommons.cancer.gov/pdc/. Drug sensitivity data were obtained from the Genomics of Drug Sensitivity in Cancer (GDSC) database through the GSCALite platform at https://guolab.wchscu.cn/GSCA/. Single-cell expression and functional analyses were performed using the TISCH and CancerSEA databases. Additional public resources used in this study included the Human Protein Atlas (HPA), TIMER2.0, UALCAN, cBioPortal, STRING, GEPIA2, Metascape, NCBI, JASPAR, and the Molecular Signatures Database (MSigDB).
