# Supplementary material for: Systematic pan-cancer analysis reveals context-dependent prognostic and immunological roles of WDR1 with divergent effects in renal and gastric cancers
Source: Front Immunol. 2026 Jun 30;17:1864405. doi: 10.3389/fimmu.2026.1864405 (PMC13366190; doi:10.3389/fimmu.2026.1864405)
Supplement: Supplementary file 1 [file DataSheet1.pdf]

Supplementary Material

Supplementary Figures

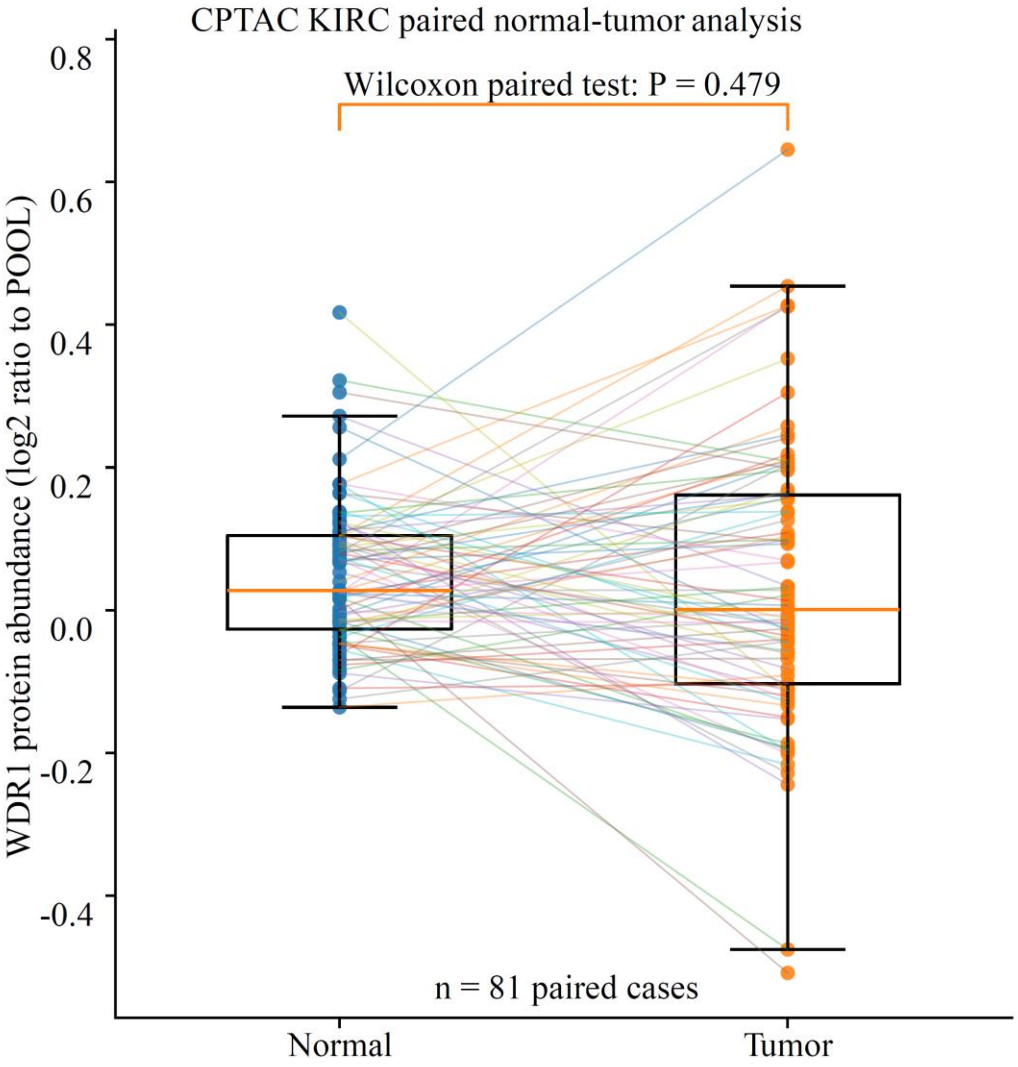

	**Normal**	**Tumor**
Mean	0.0509	0.0338

Supplementary Figure 1. CPTAC-based proteomic analysis of WDR1 in paired normal and KIRC tumor samples. WDR1 protein abundance was compared between 81 paired tumor and adjacent normal kidney tissues using CPTAC TMT10 proteomic data. Protein abundance values were presented as log2 ratios relative to a pooled reference sample (POOL). Statistical significance was assessed using the paired Wilcoxon signed-rank test.

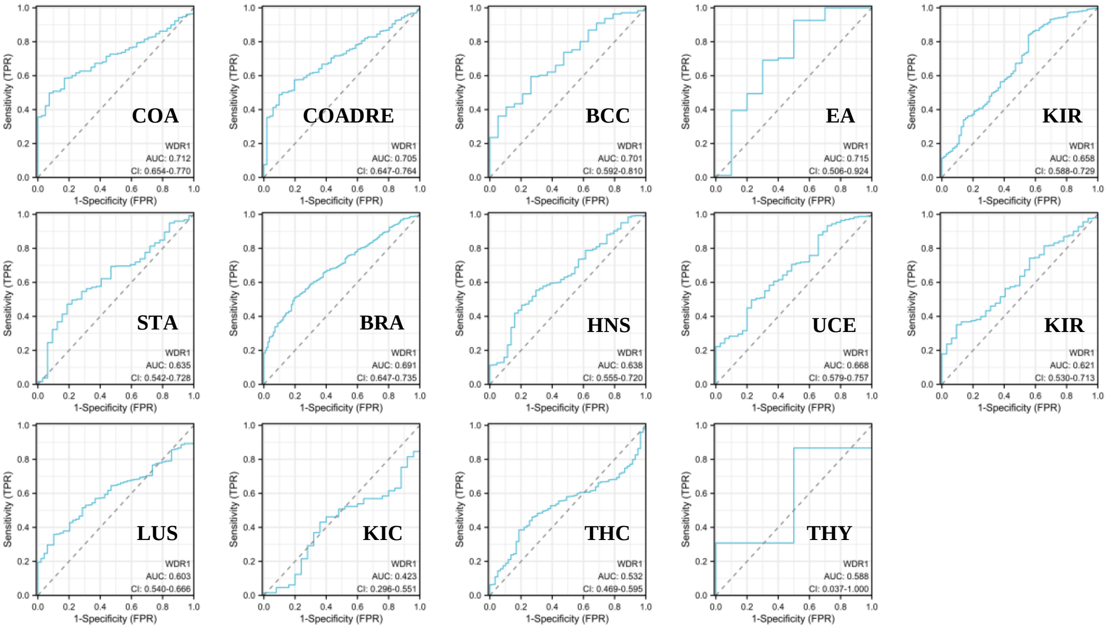

Supplementary Figure 2. Receiver operating characteristic (ROC) curve analysis of the prognostic value of WDR1 expression in pan-cancer. ROC curves were used to evaluate the predictive performance of WDR1 expression for survival outcomes in COAD, COADREAD, BRCA, ESCA, KIRC, STAD, BRAC, HNSC, UCEC, KIRP, LUSC, KICH, THCA and THYM. The area under the curve (AUC) and corresponding 95% confidence intervals (CIs) are presented.

List of Abbreviations

ACC, adrenocortical carcinoma
ADF, Actin Depolymerizing Factor
AIP1, actin-interacting protein 1
ALL, acute lymphoblastic leukemia
AUC, area under the curve
BLCA, bladder urothelial carcinoma
BRCA, breast invasive carcinoma
CAFs, cancer-associated fibroblasts
cBioPortal, cBioPortal for Cancer Genomics
CCK-8, Cell Counting Kit-8
CD8+ T cell, cytotoxic T lymphocyte
CESC, cervical squamous cell carcinoma and endocervical adenocarcinoma
CHOL, cholangiocarcinoma
CIs, confidence intervals
CNV, copy number variation
COAD, colon adenocarcinoma
COADREAD, colon and rectum adenocarcinoma
DLBC, lymphoid neoplasm diffuse large B-cell lymphoma
DNAss, DNA stemness score
DSS, disease-specific survival

EMT, epithelial–mesenchymal transition
Endo, endothelial cells
Eos, eosinophils
ESCA, esophageal carcinoma
FBS, fetal bovine serum
FDR, False Discovery Rate
fhT cells, follicular helper T cells
GBM, glioblastoma multiforme
GBMLGG, glioma
GDSC, Genomics of Drug Sensitivity in Cancer
GEPIA2, Gene Expression Profiling Interactive Analysis 2
GIST, gastrointestinal stromal tumor
GISTIC, Genomic Identification of Significant Targets in Cancer
GO, Gene Ontology
GSCA, Gene Set Cancer Analysis
GSCALite, Gene Set Cancer Analysis Lite.
GSEA, Gene Set Enrichment Analysis
GTEx, Genotype-Tissue Expression
HNSC, head and neck squamous cell carcinoma
HPA, Human Protein Atlas
HRs, hazard ratios
HSCs, hematopoietic stem cells
IC50, inhibitory concentration
IHC, immunohistochemistry
ILCs, innate lymphoid cells
JASPAR, Joint Project on the Collection and Analysis of Promoters of Eukaryotic Genes
KEGG, Kyoto Encyclopedia of Genes and Genomes
KICH, kidney chromophobe
KIPAN, pan-kidney cohort
KIRC, kidney renal clear cell carcinoma
KIRC, kidney renal clear cell carcinoma
KIRP, kidney renal papillary cell carcinoma
LAML, acute myeloid leukemia
LGG, brain lower grade glioma
LIHC, liver hepatocellular carcinoma
LUAD, lung adenocarcinoma
LUSC, lung squamous cell carcinoma
MCC, Merkel cell carcinoma
MDSCs, myeloid-derived suppressor cells
MESO, mesothelioma
MF, mycosis fungoides
MHC, major histocompatibility complex
MSI, microsatellite instability

NB, neuroblastoma
NES, normalized enrichment score
NHL, non-Hodgkin lymphoma
NK cells, natural killer cells
NPC, nasopharyngeal carcinoma
NSCLC, non–small cell lung cancer
OS, Overall survival
OV, ovarian serous cystadenocarcinoma
PAAD, pancreatic adenocarcinoma
PBMC, peripheral blood mononuclear cell
PC, pancreatic cancer
PCFCL, primary cutaneous follicle center lymphoma
PCPG, pheochromocytoma and paraganglioma
PFI, progression-free interval
PFS, progression-free survival
PPI, protein–protein interaction
PRAD, prostate adenocarcinoma
READ, rectum adenocarcinoma
RNAss, RNA stemness score
ROC, Receiver operating characteristic
SARC, sarcoma
SCLC, small cell lung cancer
SKCM, skin cutaneous melanoma
SRF, serum response factor
STAD, stomach adenocarcinoma
STES, stomach and esophageal carcinoma
STRING, Search Tool for the Retrieval of Interacting Genes/Proteins
TCGA, the Cancer Genome Atlas
TF, transcription factor
TGCT, testicular germ cell tumors
THCA, thyroid carcinoma
TIMER2.0, Tumor Immune Estimation Resource 2.0
TISCH, Tumor Immune Single-cell Hub
TMB, tumor mutation burden
TPM, Transcripts Per Million
Tregs, regulatory T cells
Tregs, regulatory T cells
TSS, transcription start site
UALCAN, A Portal for Facilitating Tumor Subgroup Gene Expression and Survival Analyses
UCEC, uterine corpus endometrial carcinoma
UCS, uterine carcinosarcoma
UVM, uveal melanoma
WDR1, WD-repeat domain 1

WT, Wilms tumor
YAP, Yes-associated protein
